# Overcoming the Biomechanical Limitations of Titanium–Zirconia Dental Implants: Rationale for a Novel Ti-PEEK-Zr Tri-Layered Concept

**DOI:** 10.3390/jfb17090469

**Published:** 2026-09-14

**Authors:** Marius Carnaru Vacaru, Corneliu Munteanu, Fabian Cezar Lupu, Grigorii Deleu, Ioana Ilinca Volocaru, Kamel Earar

**Affiliations:** 1 Mechanical Engineering Faculty, “Gheorghe Asachi” Technical University of Iasi, 700050 Iasi, Romania; drvacarumarius@yahoo.com (M.C.V.); grigorii.deleu@student.tuiasi.ro (G.D.); ioana-ilinca.volocaru@student.tuiasi.ro (I.I.V.); 2 Technical Sciences Academy of Romania, 26 Dacia Blvd., 030167 Bucharest, Romania; 3 Faculty of Dental Medicine, “Dunarea de Jos” University, 800008 Galati, Romania; kamel.earar@ugal.ro

**Keywords:** multi-material dental implant, titanium, Ti-6Al-4V, Y-TZP zirconia, polyetheretherketone (PEEK), cervical stress distribution, finite element analysis

## Abstract

**Background:** The clinical success of modern dental implants requires a balance between mechanical endurance and aesthetic integration. While titanium alloy (Ti-6Al-4V) provides a reliable load-bearing core, yttria-stabilized tetragonal zirconia (Y-TZP) is frequently preferred for the cervical collar to secure optimal peri-implant soft tissue responses. Yet, fusing these materials directly creates a structural challenge, an abrupt stiffness gradient. This discontinuity promotes localized tensile stresses within the brittle ceramic component, elevating the risk of subcritical crack initiation under oblique masticatory loads. **Methods:** To address this challenge, we conducted a narrative review to establish the rationale for a novel Ti-PEEK-Zr tri-layered concept. This approach integrates materials science and dental biomechanics to provide a theoretical framework prior to experimental testing. **Results:** The synthesized data supports the integration of polyetheretherketone (PEEK) as an intermediate compliant layer. Rather than serving as an intermediate stiffness layer, PEEK operates as a viscoelastic buffer. This functional transition zone dampens oblique forces, redistributing localized stress away from the fragile rigid–rigid junction and shielding the Y-TZP collar. The modular tri-layered configuration offers a theoretically sound mechanical hypothesis, though its clinical feasibility depends on rigorous validation that must encompass not only biomechanical performance but also biological compatibility, resistance to bacterial colonization, and long-term stability under the challenging conditions of the oral environment. The practical advantages, including manufacturability, surgical handling, and cost-effectiveness, remain to be demonstrated through future experimental and numerical studies. **Conclusions:** The Ti-PEEK-Zr multi-material concept is a biomechanical hypothesis. By functionally isolating the roles of each material, this paradigm addresses several limitations of traditional hybrid implants, providing the basis for future finite element analyses.

## 1. Introduction

### 1.1. Evolution of Dental Implant Systems

Dental implantology represents one of the most important development directions in modern dentistry, offering predictable solutions for the rehabilitation of partial and complete edentulism. The foundation of contemporary implantology is closely related to the concept of osseointegration, described by Brånemark, who demonstrated the capacity of titanium to establish a direct, stable, and functional connection with bone tissue, without the interposition of fibrous tissue at the bone–implant interface [1,2,3]. This discovery substantially changed the approach to edentulism treatment and enabled the development of modern endosseous implants.

Titanium and its alloys have become reference materials in oral implantology due to their high biocompatibility, corrosion resistance, chemical stability, and robust mechanical profile [4,5]. The evolution of dental implants has not been limited to the use of titanium as a base material, but has also included optimization of surface treatments, microtopography, the implant–abutment connection, and macrostructural design, all of which contribute to improving osseointegration and long-term stability [6,7].

Achieving osseointegration is no longer the sole clinical endpoint for contemporary dental implants. Rather, sustained functional survival depends on a finely tuned balance between tissue compatibility, mechanical durability, and optical predictability [8,9]. The long-term survival of implant restorations hinges on a dynamic set of clinical requirements. Preserving crestal bone levels and securing a competent mucosal seal represent only part of the clinical requirements. The system must also effectively dissipate occlusal forces and resist structural fatigue over time [10,11,12,13,14]. Addressing these overlapping challenges with a single material is inherently limiting. Consequently, contemporary implantology has largely embraced hybrid configurations. By strategically mixing different biomaterials, these modern assemblies can better target the mechanical, aesthetic, and biological demands of the placement site.

### 1.2. The Need for Multi-Material Approaches in Modern Implantology

Titanium remains a well-established baseline for implant fabrication. Yet, its optical density imposes a significant aesthetic limitation, especially when deployed beneath a thin gingival biotype. The gray metallic shadowing transmitted through the peri-implant mucosa is a clinical failure by modern standards, forcing an aggressive shift toward optically superior restorative materials [10].

Yttria-stabilized zirconia (Y-TZP) was integrated precisely to neutralize this visual deficit. Beyond delivering favorable optical integration, Y-TZP is chemically inert and dictates a favorable soft-tissue response [11,12,13,14,15,16,17]. This positions the ceramic as a critical asset for anterior restorations, where mucosal margin stability is the ultimate clinical endpoint [18,19]. The vulnerability, however, is strictly mechanical. Functioning as an elastic-brittle solid, zirconia severely lacks the ductility of titanium. This intrinsic brittleness increases susceptibility to crack initiation and catastrophic propagation under complex, multi-directional loading [10,20].

The structural imperative led to a direct hybridization of both materials. In these binary designs, the tough titanium core assumes the bulk of the load-bearing duty, while a zirconia collar dominates the transmucosal zone to secure the biological interface [10,11,12,13,14,15,21,22]. The primary objective is to harness the resilience of metal alongside the excellent optical properties of ceramic. Yet, this direct physical pairing engineers a severe structural penalty. Forcing two materials with drastically disparate elastic profiles into direct contact fundamentally disrupts load transfer pathways, forging a distinct biomechanical conflict right at their junction.

Addressing this interface requires rigorous empirical testing. Biological integration is a prerequisite, but it cannot override the mechanical endurance of the prosthetic complex. A viable device must execute a superior mechanical function: it must actively manage and deflect stress away from its own structural vulnerabilities. The cervical perimeter acts as the definitive critical zone, the exact coordinate where masticatory forces collide with the material transition, setting the stage for either long-term stability or catastrophic hardware failure [23,24,25].

While existing multi-material implant systems are predominantly restricted to binary configurations, alternative tri-layered approaches remain virtually non-existent or unstandardized in current clinical practice; consequently, the present conceptual model is directly juxtaposed against the established titanium–zirconia binary standard.

### 1.3. Biomechanical Limitations of Implant Systems Based on Materials with Different Stiffnesses

An implant’s biomechanical performance is never dictated by a single variable; it is governed by a strict mechanical interplay between the macro-geometry of the device, the prosthetic interface, host bone density, loading angles, and the intrinsic stiffness of the chosen materials [23,24,25,26,27]. To map these variables, finite element analysis (FEA) serves as a key computational tool. It provides more detailed insight than clinical observation alone, exposing precisely how mechanical stress disperses, or critically concentrates, throughout the peri-implant bone, across the abutment junction, and deep within the prosthetic architecture [23,24,25].

In these virtual models, the cervical region repeatedly emerges as the primary structural vulnerability. Oblique functional loads and sharp geometric transitions predictably funnel concentrated stress directly into the implant neck [28,29,30,31,32]. This cervical boundary becomes the prime determinant of long-term success. It dictates not merely whether the marginal crestal bone will resorb, but whether the hardware itself will ultimately fracture, acting as the exact nexus where severe compressive, tensile, and bending forces intersect.

In hybrid titanium–zirconia implant systems, the cervical region acquires additional importance because it may coincide with the contact area between the metallic implant body and the ceramic component. The Ti-6Al-4V alloy exhibits elastic–plastic behavior and a relatively good capacity for deformation before failure, whereas Y-TZP zirconia has predominantly elastic-brittle behavior, high stiffness, and a limited capacity for mechanical energy dissipation [5,10,20]. This difference in structural behavior may produce discontinuities in load transfer at the interface between the two materials.

The difference in elastic modulus between titanium and zirconia may generate an abrupt stiffness gradient at the structural interface. Under functional loading conditions, especially under oblique forces, this gradient may favor the occurrence of local stress concentrations near the cervical region [28,29,30,31,32]. In the case of a brittle ceramic component, such concentrations may become structurally relevant, because they may contribute to crack initiation or propagation under repeated loading conditions [20].

A primary structural limitation in binary titanium–zirconia systems is the absence of a gradual mechanical transition between the metallic and ceramic materials. A direct interface between two rigid materials with different elastic properties and failure mechanisms may amplify local stresses and may influence the long-term behavior of the ceramic component. This problem justifies the investigation of structural solutions capable of reducing stiffness discontinuity and optimizing stress distribution in the cervical region.

#### The Oral Environment: A Complex Challenge for Dental Implants

Beyond the purely mechanical considerations discussed above, dental implants function within a uniquely challenging biological and chemical environment. The oral cavity is characterized by continuous exposure to saliva, which contains electrolytes, enzymes, immunoglobulins, and a diverse microbial population. Temperature fluctuations occur during the consumption of hot and cold foods and beverages, typically ranging from approximately 5 °C to 60 °C. pH variations are equally significant; acidic dietary components and bacterial metabolites can lower the local pH to values as low as 4.0 or even lower in the presence of cariogenic biofilms. These environmental factors, combined with cyclic masticatory loading, create a complex set of demands that should be considered in any comprehensive implant design [10,20,23,24,25,27].

Saliva plays a dual role. On one hand, it provides important lubrication, buffering capacity, and antimicrobial protection. On the other hand, it serves as a medium for the transport of ions, organic molecules, and bacteria to the implant surface. The adsorption of salivary proteins to the implant surface forms a conditioning film, which subsequently mediates bacterial adhesion and biofilm formation [10,13,15]. The composition and thickness of this protein layer are influenced by the surface chemistry and wettability of the material, with hydrophobic surfaces generally tending to absorb greater quantities of protein compared to hydrophilic surfaces [10,15].

Bacterial colonization is a particularly critical concern for any implant system that features junctions or interfaces. Multi-species biofilms readily form on implant surfaces, and the presence of microgaps at component interfaces can serve as reservoirs for bacterial proliferation. The inflammatory response triggered by bacterial products can lead to peri-implant mucositis and, if untreated, peri-implantitis, which is characterized by progressive bone loss and is a leading cause of late implant failure [10,13]. Therefore, the design of any multi-material implant must prioritize not only mechanical integrity but also biological compatibility and resistance to bacterial colonization.

### 1.4. Objective and Hypothesis of the Review

Based on these considerations, this narrative review analyzes the biomechanical failures inherent to direct titanium–zirconia interfaces to establish a robust rationale for a Ti-PEEK-Zr multi-material assembly. We deliberately adopted a narrative review format specifically to integrate disparate bodies of knowledge, drawn from metallurgy, polymeric materials science, and craniofacial biomechanics, into an internally consistent explanatory schema. Examining the mechanical behavior of material interfaces as reported in the peer-reviewed record, we identify the principal architectural determinants that must subsequently inform the design of focused, tightly parameterized in vitro experiments.

Polyetheretherketone (PEEK) is a prominent high-performance thermoplastic that has found considerable use in biomedical sectors, courtesy of its excellent chemical resistance, satisfactory biocompatibility profile, and advantageous bulk mechanical behavior [33,34,35]. Within the dental field, research has turned toward PEEK for both implantological and prosthodontic indications; the polymer’s elastic modulus, which approximates that of cortical bone far more closely than do those of conventional metals or ceramics, is frequently cited as a key advantage, with reduced stress-shielding frequently postulated as a tangible clinical benefit [34,36,37,38]. More recently, the oral-applications literature has expanded to encompass detailed assessments of PEEK, underscoring not only its mechanical merits but also persistent drawbacks, chief among them its intrinsic bioinertness and the concomitant requirement for surface functionalization or adhesive augmentation [39,40,41]. Complementing these biological and physical considerations, finite-element simulations that incorporate PEEK-based abutments have reinforced the notion that this material can meaningfully alter the load-transfer landscape within implant-prosthetic complexes [42].

The primary hypothesis of this work suggests that interposing a compliant material, one whose elastic modulus is substantially lower than those of the flanking structural members, could conceivably foster a less abrupt, mechanically more compatible transition precisely at the junction where titanium abuts zirconia. Through this configuration, a redistribution of the stress field in the cervical region and a possible reduction in the maximum principal stresses developed in the ceramic component may be achieved.

By synthesizing disparate data streams regarding materials used in dental implantology, the biomechanical behavior of the cervical region, the limitations of titanium–zirconia systems, and the potential of PEEK as a transition material, this article constructs a substantiated structural hypothesis. This review does not aim to experimentally demonstrate the efficacy of the Ti-PEEK-Zr configuration, but rather to establish the rigorous, evidence-based foundation required for forthcoming numerical finite element analyses and experimental mechanical testing.

## 2. Materials and Methods

This article was designed as a narrative review oriented toward establishing the biomechanical rationale for titanium-PEEK-zirconia multi-material implant concept. This specific methodological framework was selected because evaluating a novel tri-layered configuration requires the integration of disparate scientific fields: metallurgy, polymer science, and clinical dental biomechanics. Unlike a systematic review, which strictly relies on homogeneous clinical trials that do not yet exist for this precise assembly, a narrative approach allows for synthesis of fundamental theoretical principles, material behaviors, and computational data to construct a testable structural hypothesis.

### 2.1. Literature Search Strategy

The literature search was executed in April 2026 across three primary academic databases: PubMed/MEDLINE, Scopus, and Web of Science. The search was not restricted by a start date, encompassing the foundational literature on osseointegration and classical materials science (dating from 1975) through to the most recent advancements published between 2023 and 2026. No language restrictions were applied during the initial search, though non-English articles were subsequently excluded during the screening process. The following comprehensive Boolean search string was employed: (“multi-material dental implant” OR “hybrid dental implant”) AND (“Ti-6Al-4V” or “Y-TZP zirconia” OR “polyetheretherketone” or “PEEK”) AND (“cervical stress” OR “biomechanics” OR “finite element analysis” OR “FEA”). For PubMed/MEDLINE, the search was performed using the following syntax: (“multi-material dental implant” OR “hybrid dental implant”) AND (“Ti-6Al-4V” OR “Y-TZP zirconia” OR “polyetheretherketone” OR “PEEK”) AND (“cervical stress” OR “biomechanics” OR “finite element analysis” OR “FEA”). For Scopus and Web of Science, the syntax was adapted to ((“multi-material dental implant” OR “hybrid dental implant”) AND (“Ti-6Al-4V” OR “Y-TZP zirconia” OR “polyetheretherketone” OR “PEEK”) AND (“cervical stress” OR “biomechanics” OR “finite element analysis” OR “FEA”)).

The captured literature encompassed peer-reviewed scientific articles, computational finite element analysis studies, experimental in vitro research, and governing technical standards (e.g., ISO 14801) dictating the dynamic loading of endosseous dental implants.

### 2.2. Screening and Selection Process

The initial search across all databases yielded 197 records. Following the removal of duplicate records (*n* = 42), 155 titles and abstracts were screened for relevance to the four core pillars of the review: (1) the mechanical properties of the individual materials (Ti-6Al-4V, Y-TZP zirconia, and PEEK), (2) biomechanical behavior of the cervical region, (3) limitations of the titanium–zirconia interface, and (4) the structural justification for the immediate compliant layer. Based on this initial screening, 43 records were excluded for lack of relevance. Subsequently, 112 full-text articles were assessed for eligibility. The exclusion criteria applied during this phase were conference abstracts (*n* = 23), articles not published in English (*n* = 7), and studies that did not substantively address at least one of the four core pillars of the review (*n* = 6). Following this rigorous screening process, a total of 76 peer-reviewed articles were included in the final synthesis.

### 2.3. Data Synthesis and Interpretation

All included sources were critically evaluated according to their mechanistic relevance of the investigated hypothesis. Given the narrative nature of this review and the absence of direct clinical validations for a Ti-PEEK-Zr implant system utilizing this exact structural configuration, a formal risk of bias assessment (as used in systematic reviews) was not applicable. Instead, the interpretation of the data was performed through rigorous biomechanical extrapolation, synthesizing fundamental theoretical principles, material behaviors, and computational data to construct a testable structural hypothesis.

It is important to emphasize that findings from studies involving PEEK abutments, CFR-PEEK implants, polymeric attachments, or alternative implant geometries were used solely to illustrate the generic buffering potential of such polymers and to define the critical parameters for future validation. These data were not directly extrapolated to the behavior of the proposed tri-layer implant, as no such direct evidence yet exists. Therefore, the conclusions formulated in the present review do not claim direct clinical efficacy. Rather, they serve as a conceptual substantiation and a definitive research hypothesis, identifying the critical parameters required to guide subsequent numerical simulations and experimental mechanical evaluations.

## 3. The Cervical Region of the Implant—A Critical Biomechanical Region

### 3.1. Load Transfer in Dental Implant Systems

Unlike natural dentition, which relies on the periodontal ligament to dampen and dissipate occlusal forces, an osseointegrated implant lacks this physiological shock absorber. Masticatory loads are driven directly from the prosthetic restoration through the abutment and implant body, terminating rigidly in the surrounding bone. This rigid biomechanical coupling dictates that the device’s stress distribution pattern is not merely a design feature but the primary determinant of the assembly’s long-term survival [23,24,25,43,44].

While multiple geometric and material variables govern this transfer cascade [25,26,27,28,29,30,31,32,43,44], the biomechanical chain possesses a definitive structural bottleneck, the cervical region. This is the exact nexus where loads abruptly transition from the prosthetic superstructure into the implant body and the marginal crestal bone.

This cervical zone plays a decisive role in both the preservation of the peri-implant bone and the mechanical integrity of the hardware itself. The dynamic becomes particularly critical when implants are constructed from multiple materials. In these hybrid designs, the cervical architecture frequently harbors junctions between constituents with drastically different stiffness and deformability profiles. This mechanical heterogeneity alters how forces are directed through the device, triggering non-uniform, highly concentrated stress patterns that diverge significantly from the predictable behavior of monolithic systems.

### 3.2. Influence of Oblique Loading in Biomechanical Behavior

Within the oral cavity, dental implants rarely experience purely axial loading [45,46]. Instead, mastication routinely introduces oblique and lateral force vectors, shaped by occlusal topography, jaw kinematics, functional occlusal guidance, and potentially parafunctional habits. Such off-axis loading imposes an intricate interplay of compressive, tensile, shear, and bending stresses, the cervical region bearing the brunt of these effects far more severely than under straightforward vertical loading [23,25,31,47].

The clinical importance of oblique loads lies in their generation of bending moments and uneven mechanical fields across both the implant body and the adjacent bone bed [48]. To thoroughly interrogate these potentially destructive conditions, contemporary biomechanical and fatigue-testing frameworks deliberately prioritize inclined loading regimes as worst-case clinical proxies. The ISO 14801 technical specification provides a standardized dynamic testing methodology for endosseous implants, facilitating side-by-side comparisons of differently sized or configured systems under reproducible mechanical demands [49].

When evaluating multi-material constructs, the relevance of off-axis loading takes on added significance, as bending stresses are prone to magnify localized forces in the vicinity of material junctions. In the specific case of titanium–zirconia assemblies, where the two constituents exhibit pronounced disparities in rigidity and fracture behavior, such loading patterns can fundamentally dictate the manner in which mechanical energy is transmitted into the brittle ceramic component [10,20,47].

### 3.3. Stress Distribution in the Cervical Region

Finite-element investigations consistently indicate that the cervical sector is a locus of elevated equivalent and principal stress magnitudes, a finding particularly pronounced under non-axial test conditions [23,28,29,30,31,32,44,47]. This phenomenon is attributable to the region’s anatomical and structural position, sandwiched between the prosthetic superstructure, the implant shaft, and the crestal bone, together with the inevitable geometric discontinuities inherent to that transition zone.

Commonly adopted output metrics in implant biomechanics encompass von Mises equivalent stress distributions, principal stress fields, total deformation profiles, and spatial maps of stress-concentration foci [23,24,25,44,50,51,52]. Von Mises criteria are predominantly applied to metallic members, whereas principal tensile stresses are deemed more informative for brittle materials such as dental ceramics, given their direct correlation with the likelihood of crack nucleation in tension-dominated zones [20].

Within the cervical domain, stress peaks can emerge within the implant itself, the prosthetic abutment, or the supporting marginal bone. In hybrid device architectures, this region additionally accommodates the physical boundary between different materials, thereby injecting a further layer of biomechanical intricacy into the overall mechanical response [10,20,47].

### 3.4. Factors Favoring Cervical Stress Concentration

Cervical stress concentration arises from a confluence of geometric, mechanical, and functional determinants. From a geometric standpoint, abrupt alterations in cross-sectional area, localized changes in diameter, and the specific design of the implant–abutment joint all act as significant stress raiser [28,29,30,31,32,44].

Functionally, the vectoral orientation of the applied occlusal force governs the ultimate severity of these peaks. Whereas axial loads generally promote a relatively homogeneous stress field, oblique contacts induce bending components and asymmetrical mechanical patterns that are potentially more damaging.

**Figure 1:**
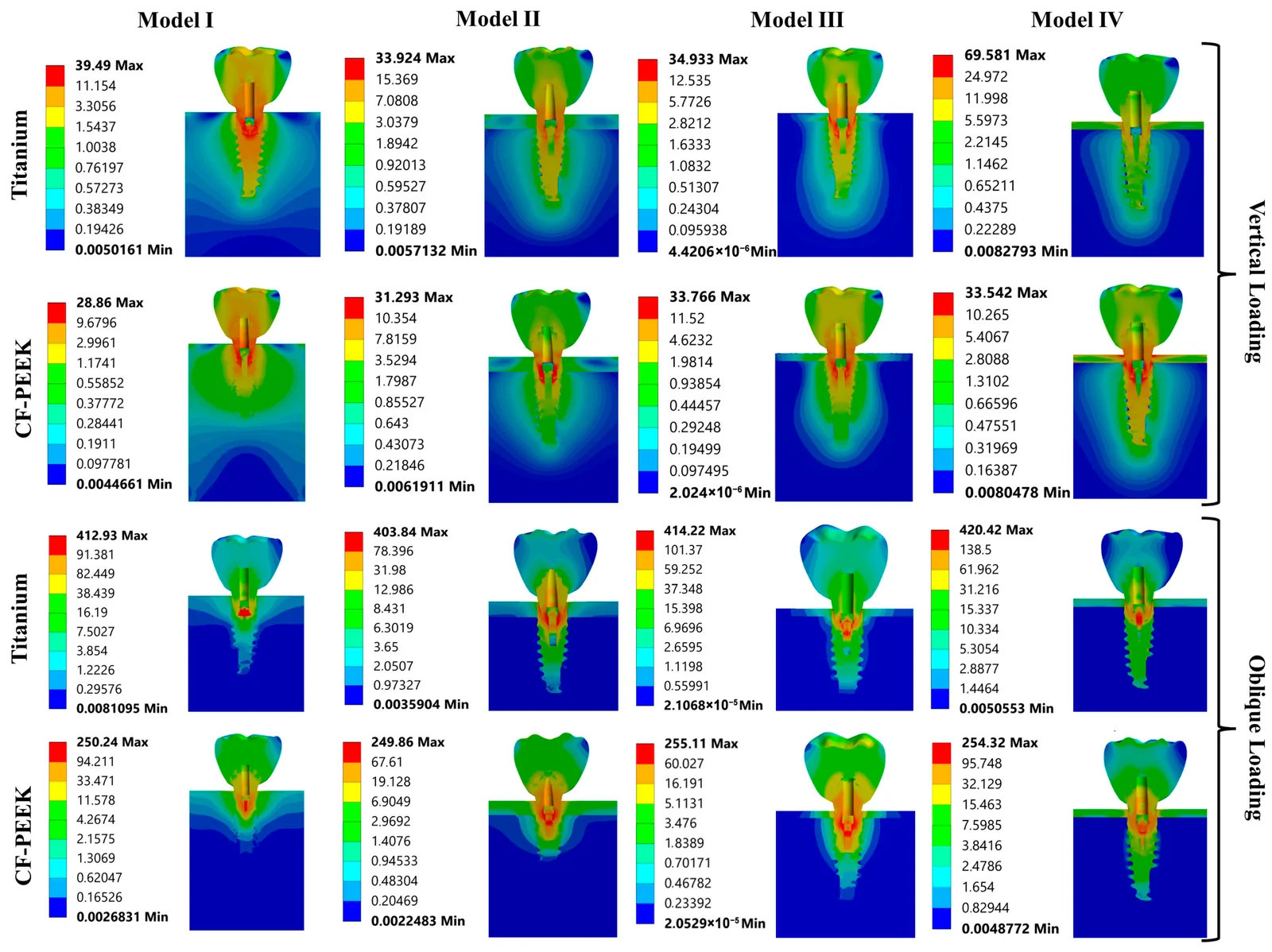
Finite element analysis mapping the severe amplification of stress concentrations under oblique loading conditions compared to axial forces (adapted with permission from Ref. [53]).

These effects are more pronounced in the cervical region, where loads are transferred to the marginal bone and where variations in geometry or structural stiffness frequently occur [23,25,31,32,44,47]. The amplification of localized stress generated by oblique trajectories, as opposed to vertical loading, is illustrated through finite element mapping in Figure 1 [53].

In multi-material systems, an additional factor is the difference in mechanical properties between the components in contact. In a titanium–zirconia interface, this fundamental mechanical mismatch inherently leads to non-uniform load transfer. The abrupt stiffness gradient at the interface may favor stress concentration in the stiffer and more brittle component, especially under oblique loading conditions [10,20,47].

These geometric, functional, and material-related factors that amplify cervical stress and dictate structural consequences are systematically synthesized in Table 1.

### 3.5. Clinical and Structural Implications of Stress Concentration

Stress concentration in the cervical region may have implications for both peri-implant tissues and the structural components of the implant assembly. At the marginal bone level, unfavorable load distributions may contribute to local micro-deformations and may influence peri-implant bone remodeling. At the level of prosthetic or implant components, concentrated stresses may promote mechanical complications such as connection loosening, component damage, or initiation of local failure mechanisms [13,25,43,44].

In the case of ceramic components, the structural implications are particularly important. Y-TZP zirconia has favorable mechanical properties for numerous dental applications, but its brittle behavior makes local tensile stresses relevant for crack initiation. Once initiated, cracks may progress under repeated loading, especially under cyclic or lateral loading conditions [20,47,54,55,56].

Therefore, mapping cervical stress concentrations is not merely descriptive. It serves as the definitive metric for quantifying structural risk. For assemblies integrating brittle ceramics at the cervical boundary, controlling this stress distribution is an essential prerequisite for long-term survivability.

### 3.6. Relevance of the Cervical Region for Multi-Material Implant Systems

In multi-material implant systems, the cervical region acquires additional biomechanical importance because it may represent the region where materials with different elastic properties, structural characteristics, and failure mechanisms meet. If a zirconia ceramic component is integrated into the cervical region of a titanium implant body, the titanium–zirconia interface overlaps with a region already predisposed to stress concentration [10,20,47].

This overlap between a biomechanically vulnerable area and an interface between materials with different stiffnesses justifies the need for a specific analysis of titanium–zirconia systems. In the absence of a gradual mechanical transition, functional loads may be transferred non-uniformly between the metallic and ceramic components, with possible amplification of stresses in zirconia.

The cervical zone must be treated as the primary structural bottleneck in multi-material implant design. Introducing a low-stiffness compliant layer, such as PEEK, establishes a plausible biomechanical mechanism to dampen the stiffness gradient and actively redistribute interfacial stresses [33,34,36,39,42]. Validating this protective mechanism now demands rigorous numerical simulation and fatigue testing.

## 4. Mechanical Properties of the Materials Involved in Multi-Material Implant Systems

### 4.1. Ti-6Al-4V Alloy Used in Dental Implantology

Titanium and its alloys occupy a central position in dental implantology due to the favorable combination of biocompatibility, mechanical strength, corrosion resistance, and osseointegration capacity [1,4,5,57]. Within implant systems, these properties justify the use of titanium as the main structural material, capable of supporting functional loads transmitted through the prosthetic superstructure and ensuring long-term mechanical stability.

The Ti-6Al-4V alloy is one of the most widely used titanium alloys in structural biomedical applications. From a metallurgical standpoint, Ti-6Al-4V is classified as a duplex α+β alloy. Aluminum preferentially partitions into and stabilizes the hexagonal α-lattice, whereas vanadium concentrates in and preserves the body-centered cubic β-phase [5,57]. The resultant grain architecture delivers an optimal balance of tensile strength, elastic rigidity, and plastic extensibility, a combination that consistently outperforms commercially pure titanium across a wide spectrum of load-bearing scenarios.

The final mechanical response of this alloy is not fixed. Rather, it is highly sensitive to compositional nuances, thermomechanical processing routes, post-fabrication heat regimens, and the resulting microstructural refinement. In the biomedical arena, its elastic modulus is conventionally cited around 105–115 GPa, and its ultimate tensile strength substantially exceeds that of unalloyed titanium [4,5,57]. This property mix renders Ti-6Al-4V particularly well-suited for implant parts that must endure multifaceted force regimes, encompassing off-axis and cyclical masticatory challenges.

One defining hallmark of this titanium grade is its unmistakable elastic–plastic operational mode. In stark contradistinction to fragile ceramics, the alloy concedes appreciable plastic flow prior to outright fracture. This intrinsic capacity for controlled yielding permits a degree of mechanical energy absorption and, crucially, attenuates the acute vulnerability to local stress raisers that plagues more brittle materials [5,57]. Within the implant context, such ductility offers a tangible safeguard; metallic members can accommodate fluctuating occlusal forces without precipitous, catastrophic collapse.

In the context of a hybrid implant, Ti-6Al-4V assumes the role of the principal structural anchor. When this metallic core is placed in direct apposition to the inflexible Y-TZP ceramic, however, an unavoidable and stark rigidity mismatch materializes at their common boundary. This mechanical incompatibility becomes particularly critical within the cervical zone, where oblique masticatory forces superimpose detrimental compression, tensile, and bending wavefronts [23,25,47]. Hence, a meaningful appraisal of Ti-6Al-4V’s suitability cannot remain confined to its standalone properties. It must forcefully pivot toward a rigorous examination of its interfacial interaction with neighboring materials.

### 4.2. Y-TZP Zirconia–Mechanical Properties and Structural Behavior

Yttria-stabilized tetragonal zirconia (Y-TZP) is a polycrystalline ceramic material used in dentistry because of its aesthetic properties, chemical stability, and biocompatibility [10,11,12,15,58,59,60]. In dental implantology, zirconia has attracted interest especially for applications in aesthetic areas, where its white color and favorable behavior of peri-implant soft tissues represent important advantages compared with metallic materials [11,12,15,61].

Structurally, Y-TZP operates as an oxide ceramic with a tetragonal phase stabilized by yttrium oxide. A critical mechanism of its mechanical behavior is transformation toughening, whereby local transformation of the tetragonal phase into the monoclinic phase around the crack tip may contribute to increased resistance to crack propagation [60,62,63]. This mechanism explains why zirconia has advantageous mechanical properties compared with many conventional oxide ceramic materials.

Y-TZP zirconia exhibits an elastic modulus typically falling between 190 and 210 GPa, coupled with impressive compressive and flexural strength values [10,59,60,63]. Yet, despite these figures, its mechanical response is fundamentally elastic-brittle, offering virtually no plastic deformation before fracture. This behavioral signature clearly sets it apart from ductile alloys like Ti-6Al-4V, which yield gradually under increasing load.

Zirconia failure is governed absolutely by crack initiation and propagation. This destructive cascade is directly triggered by microstructural defects, residual porosity, processing imperfections, low-temperature degradation, and local stress concentrations [20,59,63]. In geometrically restricted elements, such as cervical ceramic collar, these inherent vulnerabilities are critically magnified because the material completely lacks the capacity for plastic stress distribution. When integrated into hybrid systems, the extreme mechanical mismatch between Y-TZP and Ti-6Al-4V, specifically their stark contrast in elastic moduli and failure models, forces an aggressive, highly non-uniform load distribution [10,20]. Zirconia cannot be evaluated solely on its aesthetic merits; it must be rigorously managed as a fragile, stress-sensitive component highly vulnerable under oblique loading.

### 4.3. PEEK–Mechanical Properties and Characteristics Relevant to Biomedical and Dental Applications

Polyetheretherketone (PEEK) is a high-performance thermoplastic polymer used in biomedical applications due to its chemical stability, biocompatibility, resistance to degradation, and favorable mechanical properties [33]. In dentistry, PEEK has been investigated for applications in implantology, prosthetics, superstructures, abutments, and other dental components, being considered a material of interest because of its reduced stiffness compared with metallic and ceramic materials [34,36,39].

Mechanically, PEEK exhibits an elastic modulus drastically lower than both Ti-6Al-4V and Y-TZP. For unreinforced PEEK, reported values are generally approximately 3–4 GPa, closer to the range of cortical bone elastic modulus than metallic or ceramic materials used in implantology [33,34,36,39]. This property explains the interest in PEEK for applications in which the aim is to reduce extreme stiffness differences or to modify the mode of load transfer.

Unlike Y-TZP zirconia, PEEK does not exhibit elastic-brittle behavior, but rather a polymeric behavior with the possibility of controlled deformation under load. This characteristic gives it a greater capacity for partial absorption or dissipation of mechanical energy compared with brittle ceramic materials [33,39]. For this reason, PEEK can be analyzed as a compliant material in a multi-material structure, with the potential to modify local stress distribution.

Despite these buffering capabilities, PEEK presents notable clinical limitations. Its relative bioinertness, the difficulty of obtaining stable adhesion with other materials, and the need for surface treatments to improve biological or adhesive interactions are aspects discussed in the recent literature [39]. Therefore, the use of PEEK in implant systems must be carefully evaluated according to the structural role assigned to this material.

Within the proposed Ti-PEEK-Zr concept analyzed in the present review, PEEK is not proposed for the main load-bearing duties; instead, it is strategically positioned as a low-stiffness intermediate layer between the metallic core and the ceramic collar. Its potential role is to modify load transfer at the interface and to influence stress distribution in the cervical region. This hypothesis is supported by the growing interest in the use of PEEK in implant-prosthetic components and by numerical studies that have analyzed the influence of this material on stress distribution in implant assemblies [34,36,39,42].

### 4.4. Comparative Analysis of the Mechanical Properties of the Studied Materials

The comparative analysis of the mechanical properties of Ti-6Al-4V, Y-TZP, and PEEK highlights major differences in stiffness, deformation mechanism, and failure behavior. These differences are important for understanding how mechanical loads are transferred in a multi-material implant system and for justifying the need for an intermediate layer between titanium and zirconia.

Ti-6Al-4V has an elastic modulus intermediate between Y-TZP zirconia and PEEK, high mechanical strength values, and elastic–plastic behavior, which recommends it as the main structural material of the implant body [4,5,57]. Y-TZP zirconia has a significantly higher elastic modulus and high mechanical strength, but its elastic-brittle behavior makes it sensitive to local tensile stresses and microstructural defects [10,20,59,62]. These fundamental morphological disparities, which dictate the ultimate structural vulnerability of the rigid ceramic, are distinctly observable at the micro-structural level, as detailed in recent comparative SEM analysis [15].

PEEK, by contrast, has much lower stiffness and polymeric behavior, which differentiates it from both materials and makes it relevant as a compliant material in multi-material structures [33,34,36,39].

Notably, PEEK is not a material with numerically intermediate stiffness between Ti-6Al-4V and Y-TZP. Its elastic modulus is lower than that of both materials. Therefore, its potential role should not be described simplistically as an “intermediate material” in terms of elastic modulus, but more rigorously as a compliant layer or a functional transition layer capable of locally modifying load transfer between two rigid components.

The titanium core provides the necessary structural endurance, the zirconia collar ensures aesthetic and biological integration, and the PEEK layer may actively mitigate the dangers of a rigid–rigid interface. These profound mechanical discrepancies, which form the foundational rationale for the proposed Ti-PEEK-Zr concept, are systematically compared in Table 2.

## 5. Limitations of Existing Titanium–Zirconia Systems

### 5.1. Differences in Elastic Modulus and Biomechanical Implications

Titanium–zirconia implant systems have been developed with the aim of combining the structural advantages of titanium with the aesthetic and biological properties of zirconia. Titanium and its alloys, particularly Ti-6Al-4V, provide high mechanical strength, toughness, ductility, corrosion resistance, and a demonstrated capacity for osseointegration, which justifies their use as the main load-bearing material in the implant body [1,4,5,57]. In contrast, Y-TZP zirconia has important aesthetic and biological advantages, being used in implantology because of its favorable color, chemical stability, and favorable interaction with peri-implant tissues [10,11,12,15,59,60,61].

Mechanically, these two materials exhibit stark contrasts in their stiffness profiles and failure modes. A profound stiffness disparity dictates the interaction between the two principal materials. Ti-6Al-4V operates at an elastic modulus of 105–115 GPa, whereas Y-TZP zirconia enforces a massive structural rigidity of 190–210 GPa [4,5,10,58,59,60]. This marked mechanical mismatch inevitably introduces a pronounced discontinuity precisely at the metallic–ceramic junction, with direct consequences for how physiological occlusal forces are subsequently conveyed through the implant–prosthetic chain.

For devices fabricated from a single material, the overall mechanical field is governed largely by macro-geometry, local bone quality, force trajectory, and intrinsic material parameters. Once multiple materials are introduced, however, the junctions between them impose an extra layer of mechanical intricacy. Divergent elastic responses and contrasting fracture modes can provoke an uneven redistribution of forces, a situation that becomes especially acute in zones already predisposed to mechanical amplification owing to geometric constraints or functional overloading [23,28,29,30,31,32].

Thus, compelling titanium and zirconia into direct, unmediated contact within a heavily loaded region is a recipe for pronounced localized stress peaks. Ti readily accommodates applied forces through a combination of elastic and plastic strain. Zr, by contrast, affords minimal latitude for deformation prior to rupture. This fundamental asymmetry in constitutive response tends to funnel excessive mechanical energy into the ceramic component—an effect that is considerably exacerbated when loads are applied off-axis or cyclically [10,20,47,49].

### 5.2. Structural Discontinuities at the Titanium–Zirconia Interface

The junction where Ti meets Zr constitutes a distinct structural domain where two materials with fundamentally divergent mechanical behaviors converge. Biomechanically speaking, this boundary is far more than a passive partition. It operates as a high-stakes conduit that channels multiaxial forces from a yielding metallic core into a fracture-sensitive ceramic component.

In hybrid configurations, zirconia is typically deployed across the cervical or transmucosal sector, a locale already notorious for its vulnerability to mechanical concentration. Should the metal–ceramic junction coincides with this exact zone, the material discontinuity effectively superimposes itself upon a region of inherent biomechanical vulnerability [23,28,29,30,31,32,47]. Such coincidence can only exacerbate the impact of applied loads, frequently precipitating uneven mechanical fields within the overlying ceramic member.

Multiple determinants conspire to establish these structural discontinuities: the elastic modulus differential, abrupt geometric transitions, diameter changes, the quality of physical apposition between parts, and the specific assembly protocol employed. Under off-axis masticatory forces, these variables are prone to act synergistically, collectively generating tensile and flexural peaks within the vulnerable cervical sector [23,25,43,47,49].

A direct Ti–Zr interface affords no provision for a graduated mechanical transition. The leap from an elastoplastically accommodating metal to an absolutely rigid, brittle ceramic inevitably engenders an excessively steep stiffness gradient. Without an interposed mediator to temper this abrupt transition, the structural integrity of the ceramic partner remains perpetually compromised.

### 5.3. Stress Concentration in the Cervical Region

The implant’s cervical segment is widely acknowledged as a high-risk territory for stress accumulation, particularly when the direction of loading deviates from the long axis. Computational models routinely confirm that peak equivalent and principal stress values cluster around the implant neck, the crestal bone interface, and the vicinity of joints or material transitions [23,28,29,30,31,32,47,49].

Within Ti–Zr hybrid designs, this cervical stress concentration assumes an even greater significance, since the cervical zone is precisely where the metallic core and the ceramic ferrule typically meet. A site already primed for sophisticated force interactions additionally becomes the very plane across which the fundamental stiffness inequity between materials is most acutely felt.

Off-axis forces are a major driver of this phenomenon. In contrast to vertical loads, which typically foster a relatively balanced distribution, oblique contacts introduce bending couples and asymmetrical mechanical patterns. Such multifaceted loading exposes the cervical collar to a concurrent onslaught of compressive, tensile, and flexural demands, inevitably cultivating pockets of excessive local strain [43,47,49].

In ductile metals, localized peaks can be partially alleviated through incipient plastic flow. Zirconia, however, offers no such safety valve, given its essentially elastic-brittle response. The stresses that arise within the ceramic collar are more critical from a structural standpoint than those occurring in the metallic core [10,20,59,62]. This inescapable divergence dictates that merely pairing Ti with Zr is an insufficient guarantee of long-term biomechanical reliability. The exact design of the junction and the modality of force transmission become paramount determinants of the whole assembly’s structural fidelity.

### 5.4. Risk of Crack Initiation and Propagation in Zirconia

Y-TZP boasts mechanical credentials that surpass those of many traditional dental ceramics, a distinction largely attributable to its transformation-toughening capability. This specific micro-mechanism endows the material with enhanced fracture toughness relative to other oxide-based alternatives [60,61,62,63]. Yet, despite this advantage, Y-TZP remains fundamentally brittle; its eventual failure is still dictated by the classic sequence of crack nucleation followed by subcritical or critical growth [20,59,62].

The presence of any local stress raiser, be it an abrupt geometric change, a microstructural flaw, retained porosity, or an ill-conditioned contact patch, can substantially elevate the hazard of crack inception. This risk is particularly pronounced in slender ceramic sections or in regions where tensile excursions are magnified by off-axis or cyclic functional demands [20,47,49,62].

At the level of the zirconia cervical collar, cracks may be initiated in areas where the principal stresses exceed the local strength capacity of the material. Once initiated, they may propagate under repeated loading, especially if the stress distribution remains concentrated in the same region [20,44,47,63,64]. Thus, aggressively mitigating localized stress within the zirconia collar becomes a critical structural priority.

Low-temperature degradation (aging) represents an additional well-documented factor that can compromise the mechanical properties of Y-TZP in the humid oral environment [20,63]. Recent studies on two-piece zirconia oral implant systems subjected to artificial loading and hydrothermal aging highlight the current interest in evaluating the fracture resistance of these components under conditions close to functional loading [63].

The primary structural limitation of binary titanium–zirconia systems lies not just in material mismatch itself, but in forcing this severe mechanical conflict precisely at the implant’s most vulnerable biomechanical bottleneck. The association of a rigid–rigid interface with the cervical region of the implant may create conditions favorable to stress concentration and crack initiation in the ceramic component. This limitation justifies the investigation of mechanical transition solutions between titanium and zirconia, capable of modifying stress distribution and reducing local loads in zirconia.

### 5.5. Engineering Challenges for Future Development

Beyond the biomechanical limitations discussed above, the translation of a multi-material implant concept into a functional clinical device presents additional engineering challenges. Two critical aspects that must be addressed in future design and validation phases are:

1. Insertion Torque Management: Achieving primary stability requires the application of rotational forces of 35–50 Ncm. In a tri-layered configuration, the surgical driver must engage the titanium core directly to avoid transmitting shear forces through the compliant PEEK layer. This design consideration is detailed in Section 7.1.4 [65].
2. Prevention of Bacterial Microleakage: The presence of two distinct material interfaces (Ti-PEEK and PEEK-Zr) at the sub-crestal level introduces potential pathways for bacterial infiltration. Future designs must prioritize manufacturing tolerances and adhesive protocols to maintain a hermetic seal, thereby preserving the biological advantages of the ceramic component. This aspect is discussed in Section 7.1.3 [66].

### 5.6. Long-Term Interface Integrity and Fatigue Resistance

Beyond the immediate challenges of insertion torque and bacterial microleakage, the long-term clinical success of a multi-material implant concept depends critically on the durability of its material interfaces. The Ti-PEEK and PEEK-Zr junctions will be subjected to millions of cyclic loading cycles over the lifetime of the implant. Under such repeated functional demands, several degradation mechanisms should be considered.

The adhesion between PEEK and the adjacent rigid materials (Ti and Zr) must withstand not only static loads but also cyclic shear and tensile stresses. Delamination, the progressive separation of layers at the interface, could occur if the interfacial bond strength is insufficient or if fatigue damage accumulates over time. Recent advances in PEEK surface functionalization, including plasma treatment, sandblasting, and the application of adhesive primers containing methyl methacrylate, have demonstrated improved interfacial bond strengths [67]. However, the long-term stability of these modified interfaces under cyclic loading in a humid oral environment remains an area requiring further investigation.

At the Ti-PEEK interface, micromovements (fretting) under cyclic loading could generate wear debris, potentially triggering an inflammatory response in the peri-implant tissues. PEEK is known for its favorable wear characteristics compared to many polymers, but the specific wear behavior of the Ti-PEEK and PEEK-Zr couples under simulated masticatory loading has not been systematically characterized. Future tribological studies should evaluate wear rates, debris generation, and the potential biological response to any released particles [33,68].

PEEK, as a thermoplastic polymer, exhibits time-dependent viscoelastic behavior. Under sustained or cyclic compressive loading, the material may undergo creep deformation, potentially reducing the effective thickness of the interlayer and alter the stress distribution over time. Similarly, cyclic loading may induce fatigue damage within the PEEK layer itself, leading to microcracking and loss of mechanical integrity [33,69]. The magnitude of these effects will depend on the specific PEEK grade, the applied load magnitude, the loading frequency, and the temperature and humidity of the environment.

Interface Characterization: To address these concerns, future experimental validation must include not only global strength and fatigue testing (as discussed in Section 7.5 and Section 8.2) but also detailed post-test characterization of the interfaces. Scanning electron microscopy (SEM) with energy-dispersive X-ray spectroscopy (EDS) should be employed to examine the interfaces for evidence of delamination, microcracking, wear debris, or chemical degradation. Surface profilometry can quantify wear depth and roughness changes. These analyses are important to confirm that the interfaces can maintain their structural integrity over the intended service life of the implant.

### 5.7. Biological and Environmental Considerations

The mechanical focus of this review should not obscure the fundamental biological requirements for dental implant success. The proposed Ti-PEEK-Zr concept must be evaluated not only in terms of stress distribution but also with respect to its biological performance within the oral environment.

The titanium core of the proposed design will be in direct contact with bone tissue, which is appropriate given titanium’s well-documented capacity to support osseointegration through a stable biological interface [1,4,5]. However, the PEEK layer, as it is positioned sub-crestally, could potentially be exposed to bone tissue if marginal bone resorption occurs over time. PEEK is known to be bioinert and has limited osseointegration potential without surface modification [33,39]. This limitation has been addressed in recent research through surface functionalization strategies, including plasma treatment, sandblasting, and coating with bioactive materials such as hydroxyapatite or titanium [67]. While these approaches show promise, their long-term stability and effectiveness in the oral environment require further investigation.

The transmucosal region of a dental implant is critical for establishing a biological seal that prevents bacterial penetration. The success of the epithelial attachment depends on the surface properties of the material in contact with the gingival tissues [13,15]. The Y-TZP zirconia collar, which is intended to be the only component exposed to the transmucosal environment, has demonstrated favorable soft tissue responses compared to titanium in some studies [10,11,12,15]. However, the quality of the epithelial seal around zirconia and the potential for bacterial colonization at the zirconia-soft tissue interface remain areas of active investigation.

The surface chemistry, roughness, and wettability of the implant materials significantly influence protein adsorption, bacterial adhesion, and biofilm formation. Y-TZP zirconia has a relatively hydrophilic surface compared to many polymers, which may promote soft tissue integration, although the relationship between wettability and bacterial adhesion is complex and context-dependent [39]. The PEEK layer, if exposed to the oral environment (which is not intended in the current design), would present a hydrophobic surface that is susceptible to bacterial colonization [67]. Therefore, the design must ensure that the PEEK layer remains sub-crestal and that the zirconia collar is optimized to promote epithelial attachment while minimizing bacterial adhesion.

While titanium and zirconia are generally resistant to corrosion in the oral environment, the presence of multiple materials introduces the risk of galvanic corrosion if the materials are in direct electrical contact. The PEEK layer, being an electrical insulator, would electrically isolate the titanium and zirconia components, potentially mitigating this risk. However, the long-term stability of the PEEK material in the presence of saliva, temperature fluctuations, and pH variations must be confirmed through appropriate testing, including thermocycling and immersion in artificial saliva [68].

## 6. PEEK as a Biomechanical Transition Material

### 6.1. The Role of PEEK as a Functional Compliant Layer

In multi-material implant systems, the biomechanical behavior of the assembly is influenced not only by the individual properties of the materials used, but also by how these materials interact at structural interfaces. In a direct titanium–zirconia association, the aforementioned biomechanical differences generate a severe structural discontinuity at the contact area [4,5,10,20,58,59,60,61,62].

The concept of a stiffness gradient refers to the introduction of a progressive mechanical transition between materials with different elastic properties, aiming to reduce abrupt variations in stiffness and influence the mode of load transfer. In a strict sense, a classical stiffness gradient involves a progressive transition from one elastic modulus to another. However, in the case of the Ti-PEEK-Zr system, PEEK should not be interpreted as a material with numerically intermediate stiffness, because its elastic modulus (approximately 3–4 GPa) is considerably lower than that of both titanium and zirconia [33,34,36,39].

Therefore, instead of acting as an intermediary in a classic stiffness gradient, the PEEK layer in our proposed design functions as a functional compliant layer, an elastic buffer, or a mechanical decoupling zone. Its primary role is not to provide a linear transition of elastic moduli, but to dissipate localized strain energy through controlled viscoelastic deformation. This decouples the rigid Ti-Zr junction, preventing the direct transmission of concentrated tensile stresses into the brittle ceramic collar.

Viewed from this perspective, PEEK serves primarily as a functional buffer, its role encompassing:

- The attenuation of dynamic load peaks
- The accommodation of limited viscoelastic strain
- The favorable rerouting of mechanical trajectories across the implant-collar interface

Such a buffering intervention is particularly consequential in hybrid devices, because the cervical sector is intrinsically prone to force concentration, especially when occlusal contacts deviate from the vertical axis [23,28,29,30,31,32,43,47,49]. Interposing this compliant stratum between the metallic body and the ceramic ferrule provides a clear structural advantage: the abruptness of a rigid–rigid apposition may be measurably mitigated, yielding a more evenly distributed stress field within the overlying ceramic component.

It is important to emphasize that this compliant layer is not a guaranteed improvement. 

### 6.2. Capacity for Stress Redistribution

The efficacy of stress reallocation within a multi-material construct is governed by a confluence of parameters: the relative elastic mismatch between layers, the global geometry of the device, the physical intimacy of the contacting surfaces, the dimensional thickness of any interposed medium, and the vectorial orientation of the applied forces. Clinically, off-axis masticatory loads are particularly influential, as they generate flexural moments and asymmetrical mechanical fields whose peak levels are consistently found within the cervical perimeter [23,28,29,30,31,32,43,47,49].

When Ti and Zr are placed in direct apposition without an intervening medium, occlusal forces travel across an unmediated metallic–ceramic path. This arrangement frequently induces pronounced stress elevations within the zirconia, notably in regions where the stiffness gradient coincides with geometric contractions or where tensile and bending contributions overlap [10,20,44,47,63]. Given zirconia’s severely restricted ability to undergo inelastic deformation, these concentrated loads acquire clinical significance as potential nucleation sites for critical flaws and their subsequent extension.

The insertion of a PEEK interlayer has the potential to mitigate this unfavorable load transfer mechanism. Due to its reduced elastic modulus and polymeric behavior, PEEK may function as a compliant zone between the metallic and ceramic materials [33,34,36,39]. This zone may accommodate part of the local deformation and may contribute to reducing the abrupt transmission of loads toward the rigid and brittle component. The influence of PEEK on stress distribution is quantitatively presented in Table 3, which compares von Mises stress values between monolithic titanium and PEEK-integrated models under oblique loading [42]. While the abutment stress decreased by 13.1%, the stress in the fixation screw and implant fixture increased by 53.9% and 158.6%, respectively, highlighting that the addition of PEEK substantially alters the load transfer pathways within the implant-prosthetic assembly.

Recent data in the literature support the interest in polymeric materials for modifying stress distribution in implant-prosthetic assemblies, but they also indicate the need for cautious interpretation. Numerical analyses of implant systems including polymeric materials have shown that these materials may modify stress distribution, but they may also generate increased deformation or critical areas in certain components, depending on geometry, material, connection type, and loading direction [42,53,69].

Therefore, the effect of PEEK should not be presented as a clinical certainty, but as an argued biomechanical hypothesis. Its structural role is not to replace titanium as the load-bearing material or zirconia as the aesthetic cervical material, but to modify the mechanical interface between the two components. This approach may be particularly relevant for reducing local loads in zirconia, where tensile stresses and stress concentrators may have critical effects on fracture behavior [20,59,63,64].

### 6.3. Relevant Biomedical and Dental Applications

PEEK is a high-performance thermoplastic polymer used in biomedical applications due to its chemical stability, biocompatibility, resistance to degradation, and favorable mechanical properties [33]. In the medical field, interest in PEEK has been supported by its use in orthopedics, traumatology, and spinal surgery, where its lower stiffness compared with metallic materials may represent an advantage in certain structural contexts [33].

Within the dental field, PEEK has been the subject of extensive exploration across a broad spectrum of indications: oral implantology, fixed and removable prosthodontics, abutment fabrication, superstructure frameworks, CAD/CAM-generated restorations, and diverse implant-prosthetic attachments [34,36,39,41]. Its distinctive material signature, characterized by a notably low elastic modulus, reduced density, excellent chemical inertness, and a mechanical response that diverges sharply from both metallic and ceramic counterparts, collectively accounts for the marked surge in research attention directed toward this polymer across multiple investigative streams within contemporary dentistry.

Nevertheless, the translation of PEEK into routine oral practice is not devoid of inherent challenges. Its relative bioinertness, the difficulty of obtaining predictable adhesion with other materials, and the need for surface modification are aspects consistently discussed in the literature [34,36,39]. These limitations are important because, in a multi-material system, the performance of the assembly depends not only on the bulk properties of PEEK, but also on the behavior of its interfaces with titanium and zirconia.

**Figure 2:**
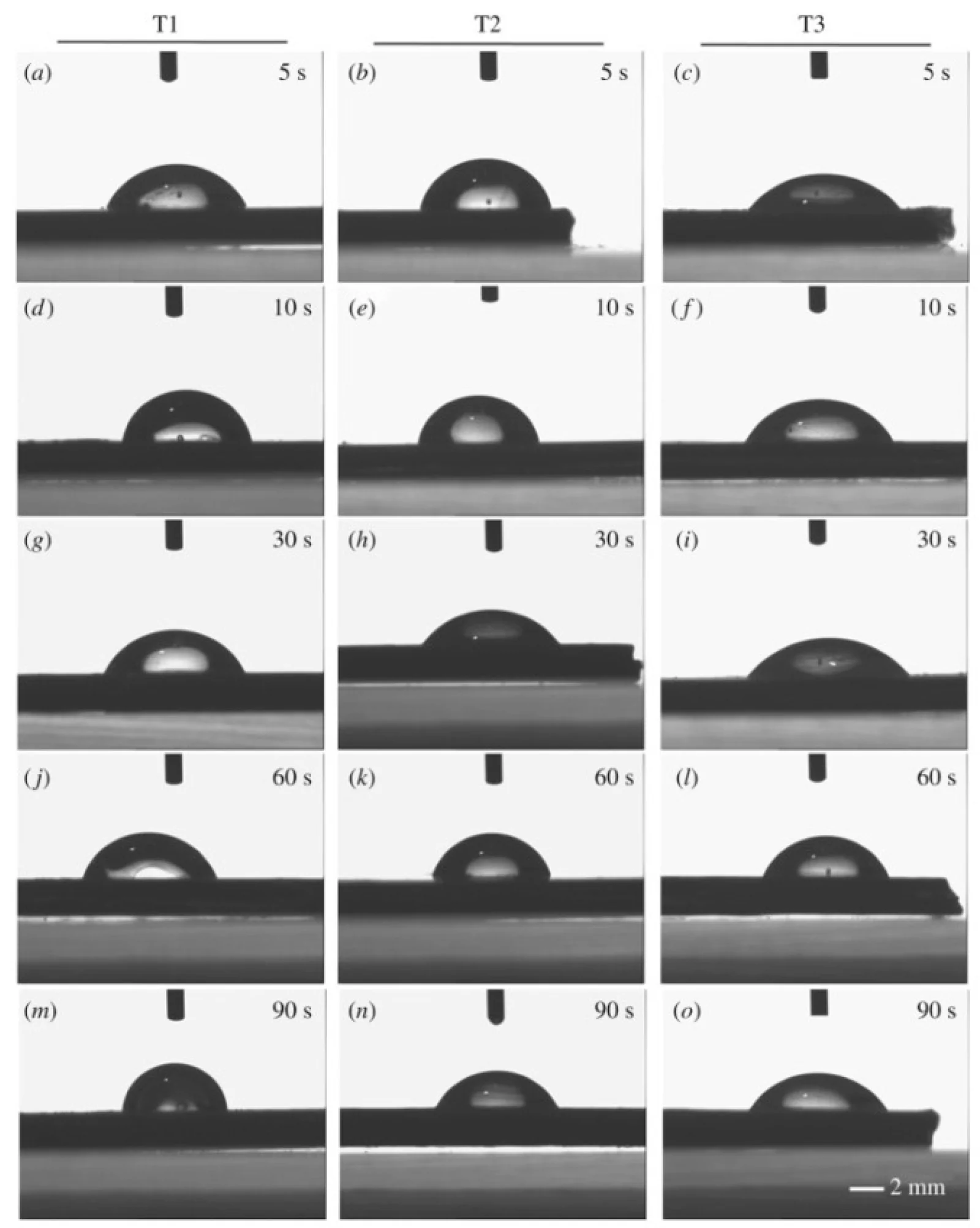
Time-resolved contact angle analysis demonstrating the dynamic optimization of PEEK surface hydrophilicity. Columns represent distinct surface modification protocols (T1–T3), while rows detail the progressive fluid spreading over time: 5 s (**a**–**c**), 10 s (**d**–**f**), 30 s (**g**–**i**), 60 s (**j**–**l**), and 90 s (**m**–**o**). Scale bar: 2 mm. This progression indicates the customizable bio-affinity of the polymer (adapted with permission from Ref. [39]).

Recent studies on advanced PEEK surface modifications indicate a critical pivot toward optimizing interfacial behavior, hydrophilicity, and cellular adhesion [67]. The efficacy of these targeted chemical treatments in dynamically neutralizing polymeric bio-inertness is demonstrated by the time-resolved contact angle measurements in Figure 2 [39]. These directions are relevant for oral applications, because they may contribute to overcoming the biological and adhesive limitations of PEEK, especially when the material is used in regions where interaction with tissues or other structural components is critical.

Within the proposed Ti-PEEK-Zr architecture, PEEK must be evaluated exclusively through its mechanical utility. If positioned between the titanium implant body and the zirconia ceramic collar, the material may function as an intermediate layer with reduced stiffness, capable of influencing load transfer in the cervical region. This use is distinct from situations in which PEEK or CF-PEEK are proposed as main implant materials [34,53].

### 6.4. Arguments for Using PEEK in Multi-Material Implant Systems

The main argument for using PEEK in a Ti-PEEK-Zr multi-material implant system derives from the need to reduce the negative effects of a direct titanium–zirconia interface. As discussed above, the direct association between a ductile metallic material and a rigid, brittle ceramic can create a mechanical discontinuity in the cervical region, an area already predisposed to stress concentration [10,20,23,28,29,30,31,32,47,49].

In this configuration, titanium plays the role of the main load-bearing element, providing mechanical strength to the implant body and transferring loads to the bone environment. Zirconia offers aesthetic and biological advantages in the cervical or transmucosal region, but its brittle behavior requires careful control of local stresses [10,11,12,15,20,59,60,61,62,63,64]. PEEK may complement this assembly by introducing a functional transition zone between the two materials.

Biomechanically, deploying an intermediate compliant layer yields three potential advantages. First, it physically dampens the collision at the rigid–rigid Ti-Zr boundary. Second, it facilitates controlled local viscoelastic deformation, actively redistributing applied loads. Third, it may suppress the maximum tensile stresses forced into the ceramic collar during oblique functional cycles.

At the same time, a prudent reading of current evidence cautions that the incorporation of polymeric interlayers is by no means a guaranteed improvement. While a lower elastic modulus can, under appropriate geometric and loading conditions, beneficially alter the overall mechanical field, it simultaneously carries the inherent risk of accentuating localized creep or adversely modulating the cyclic fatigue response of adjacent structural members [53,69]. This caveat assumes particular relevance for the Ti-PEEK-Zr proposition: the inclusion of PEEK cannot be presumed intrinsically advantageous; rather, its putative merits must be rigorously substantiated through both computational simulations and controlled in vitro mechanical verification.

In summary, these considerations position PEEK as a strategically attractive candidate for engineering a mechanical transition between the metallic core and the ceramic collar. Its intended function is not to supplant the established materials, but rather to refine the mechanical interplay between them precisely within the most vulnerable sector of the implant system. This approach supports the rationale for the Ti–PEEK–Zr concept as a research direction oriented toward reducing cervical stress concentration and improving the structural behavior of multi-material implant systems.

However, validation of this concept requires a staged approach. The first stage should include numerical finite element analysis to identify stress concentration areas and to compare configurations with and without an intermediate layer. The second stage should include experimental mechanical testing, evaluating behavior under static and cyclic loading. The third stage may include analysis of interfaces through morphological and structural methods, to evaluate contact continuity, possible defects, and local failure mechanisms. Only by correlating these levels of analysis can it be established whether the PEEK layer effectively contributes to optimizing the biomechanical behavior of the Ti-PEEK-Zr assembly.

### 6.5. Limitations and Challenges of PEEK in the Oral Environment

While the mechanical benefits of PEEK as a compliant buffer have been discussed extensively, it is important to critically evaluate the limitations and challenges associated with its use in the oral environment.

PEEK is a bioinert polymer that does not actively promote bone formation or integration [33,39]. In contrast to titanium, which supports osseointegration through a well-characterized biological interface, PEEK typically elicits a fibrous encapsulation response. This is not problematic when PEEK is used as an interlayer between titanium and zirconia, as it is not intended to be in direct bone contact. However, if marginal bone resorption exposes the PEEK layer over time, the lack of osseointegration could compromise implant stability. Surface modification strategies, including plasma treatment and coating with bioactive materials, have been explored to address this limitation [67].

PEEK is a hydrophobic material with low surface energy, which tends to promote greater absorption of salivary proteins, potentially influencing subsequent bacterial adhesion [39]. Bacterial colonization on PEEK surfaces could lead to inflammatory responses and peri-implantitis if the material is exposed to the oral cavity. In the proposed design, the PEEK layer is intended to be positioned sub-crestally, minimizing its exposure to the oral cavity, as the layer is positioned sub-crestally. However, this design feature introduces additional complexity in terms of manufacturing tolerances and interfacial seals.

PEEK exhibits viscoelastic behavior, which means that its mechanical properties are dependent on the loading rate and temperature [33,67]. Under sustained loading, PEEK undergoes creep, which could reduce the effective thickness of the interlayer and alter the stress distribution over time. Similarly, cyclic loading can induce fatigue damage, leading to microcracking and loss of mechanical integrity. These effects must be rigorously evaluated through long-term fatigue testing under conditions that simulate the oral environment.

The stability of PEEK in the oral environment has not been exhaustively studied. Exposure to saliva, which contains enzymes and reactive oxygen species, could potentially alter the surface chemistry of PEEK over extended periods [68]. Similarly, temperature fluctuations and pH variations could accelerate degradation processes. The combination of environmental aging and cyclic loading represents a worst-case scenario that must be addressed in future validation studies.

## 7. Rationale for the Ti-PEEK-Zr Concept

### 7.1. Proposed Conceptual Model and Design Specifications

Driven by the inherent structural failures of binary titanium–zirconia systems and the irreconcilable material profiles discussed previously, we conceptualize a tri-layered functional architecture: a Ti-6Al-4V core, an intermediate PEEK compliant layer, and a Y-TZP cervical collar. This model aims to combine the mechanical advantages of titanium with the aesthetic and biological properties of zirconia, using PEEK as a biomechanical transition element between the two materials.

#### 7.1.1. Geometric Configuration and Material Distribution

In this configuration, the Ti-6Al-4V implant body serves as the primary load-bearing element, responsible for receiving functional loads and transferring them to the peri-implant bone tissue. The choice of titanium is justified by its mechanical strength, toughness, elastic–plastic behavior, corrosion resistance, and favorable clinical history in oral implantology [1,4,5,57].

The Y-TZP zirconia collar is proposed for the cervical or transmucosal region, where aesthetic advantages, chemical stability, and favorable behavior of soft tissues are clinically relevant [10,11,12,15,59,60,63]. However, the elastic-brittle behavior of zirconia requires careful control of local stresses, especially under oblique or cyclic loading conditions [20,47,49,62,64].

The intermediate PEEK layer is introduced with a functional role, not as the main load-bearing element. Due to its reduced elastic modulus and polymeric behavior, PEEK may function as a compliant layer between titanium and zirconia, with the potential to modify load transfer in the cervical region [33,34,36,39,42,53,67,69].

#### 7.1.2. Anatomical Position, Geometry, and Dimensional Specifications

The PEEK layer will be positioned at the cervical region, specifically at the transition zone between the implant body and the abutment, serving as an interposed collar. The layer is conceptualized as a circumferential ring, interposed between a reduced-diameter Ti-6Al-4V core and a sleeve-like Y-TZP collar. This circumferential geometry ensures uniform stress distribution around the entire implant perimeter.

For the initial phase of numerical validation (finite element analysis), we propose a nominal PEEK layer thickness of 0.5 mm to 1.0 mm. This dimension represents a manufacturable scale that can provide a significant mechanical buffer without compromising the overall diameter of the implant (maintaining a minimum core diameter of 3.0 mm for standard 4.0 mm implants). A parametric FEA study is planned to systematically evaluate the influence of varying thicknesses on stress distribution, identifying the optimal balance between buffering capacity and structural stability.

Importantly, the PEEK layer will be located sub-crestally, and only the biocompatible Y-TZP collar will be exposed to the transmucosal environment and soft tissues. This design choice minimizes the risk of bacterial colonization on the polymer surface and preserves the aesthetic advantages of zirconia at the mucosal margin.

**Figure 3:**
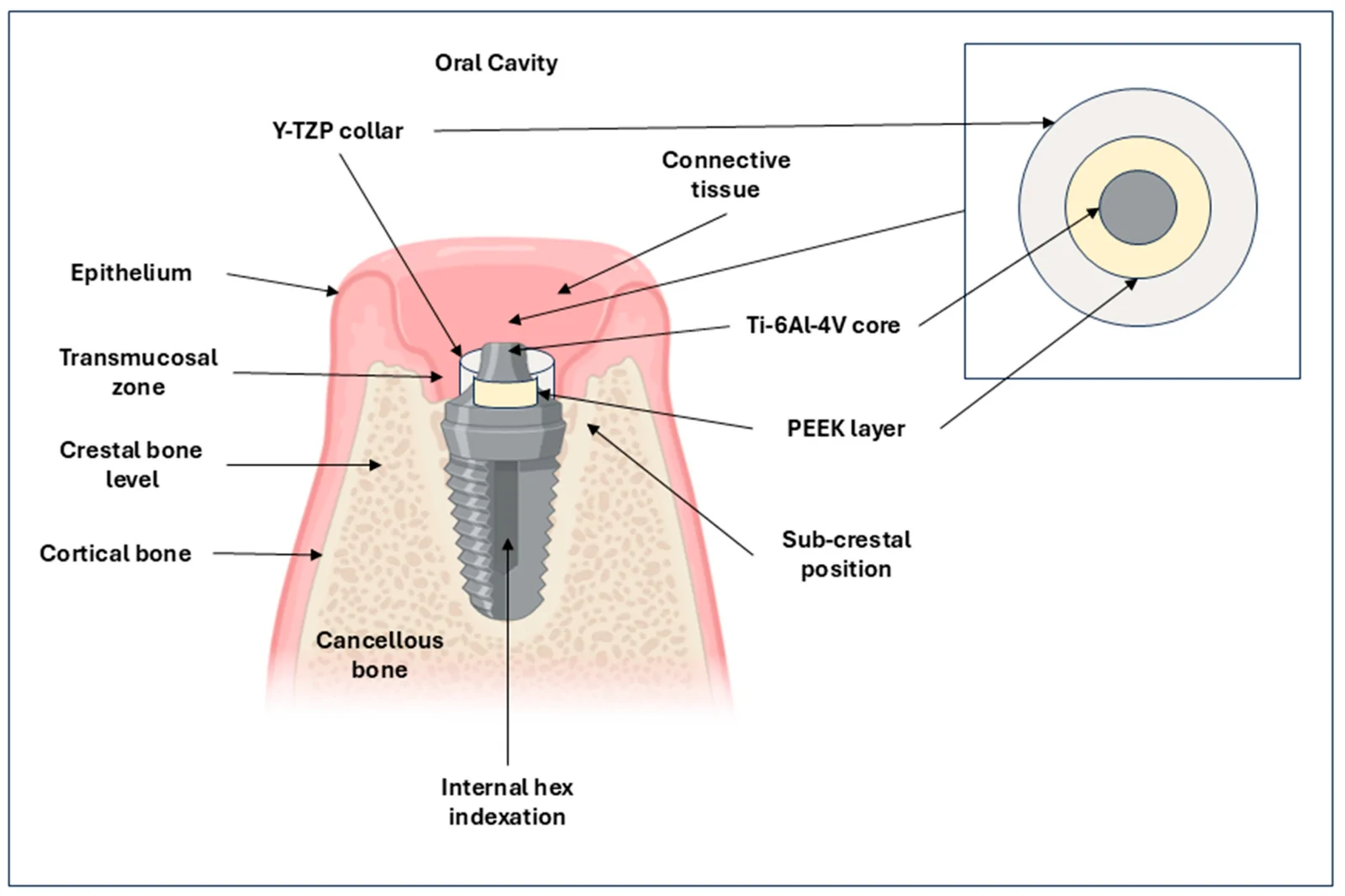
Schematic cross-sectional illustration of the proposed Ti-PEEK-Zr tri-layered implant architecture showing the anatomical relationships between the implant components and the surrounding oral tissues. The Ti-6Al-4V core (grey) is positioned within the bone; the PEEK layer (yellow) is located sub-crestally as a circumferential ring between the titanium core and the zirconia collar; and the Y-TZP zirconia collar (white) is exposed to the transmucosal environment, interfacing with the epithelium and connective tissue (pink). The design ensures that the insertion torque is transmitted exclusively through the titanium core via the internal hex indexation, while the PEEK and zirconia layers function as passive sleeves.

The anatomical relationships between the proposed implant components and the surrounding oral tissues are as follows. From top to bottom, the oral cavity is situated above the gingival margin. The gingiva (mucosa) covers the alveolar bone and is composed of two distinct layers: an outer epithelial layer (facing the oral cavity) and an underlying connective tissue layer. The alveolar bone comprises a dense cortical layer at the surface and a porous cancellous bone core in the interior. The Ti-6Al-4V implant body is positioned within the bone, extending from the apical region to the cervical level, and is in direct contact with bone tissue along its entire intraosseous length. The PEEK layer is positioned sub-crestally, meaning it is located below the crestal bone level and is not exposed to the oral cavity, connective tissue, or epithelium. It forms a circumferential ring interposed between the reduced-diameter titanium core and the outer ceramic collar, at the transition zone between the implant body and the abutment. The Y-TZP zirconia collar is positioned above the PEEK layer and is the only component exposed to the transmucosal environment. It interfaces with the epithelium and connective tissue, providing aesthetic and biological integration at the soft tissue level. The implant–abutment connection is internal and conical, with a hexagonal indexation feature machined directly into the titanium core for surgical driver engagement. This design ensures that the insertion torque is transmitted exclusively through the titanium core, while the PEEK and zirconia layers function as passive sleeves that are not subjected to rotational shear forces as schematically illustrated in Figure 3.

#### 7.1.3. Interface Establishment and Manufacturing Approach

The physical realization of the Ti-PEEK-Zr concept presents specific engineering challenges, particularly regarding the stability of the two critical interfaces. The Ti-PEEK interface will be established through a combination of mechanical interlocking and chemical adhesion. Mechanical interlocking will be achieved via micro-retentive grooves or a macro-retentive undercut geometry on the titanium core surface. This will be complemented by chemical functionalization of the PEEK surface using an adhesive system specifically designed for PEEK, such as a primer containing methyl methacrylate, as described in the recent literature [67]. The PEEK-Zr interface will be established through surface roughening of the PEEK (e.g., via sandblasting or acid etching) combined with a precision press-fit assembly, potentially augmented by a suitable adhesive interlayer.

The intended manufacturing approach is subtractive manufacturing (5-axis CNC machining for the Ti-6Al-4V core and the Y-TZP collar, and precision milling for the PEEK layer), followed by assembly. Achieving a high-precision, fluid-tight interface will require micrometric tolerances (≤±5 µm), which is achievable with modern CNC machining technologies. This level of precision is critical to minimize microgaps and potential pathways for bacterial microleakage [66].

##### Joining Methods and Their Limitations

The physical realization of the Ti-PEEK-Zr concept depends critically on the joining methods employed to establish the two interfaces. Several approaches have been investigated in the literature for joining PEEK to metals and ceramics.

Mechanical interlocking relies on macro- or micro-retentive features on the titanium surface, such as grooves, undercuts, or threads, that physically anchor the PEEK layer. Mechanical interlocking offers the advantage of being simple and reliable, but it may create stress concentrations at the interface and may not provide a fluid-tight seal. The precision of the mechanical interlocking features and the quality of the press-fit assembly are critical for minimizing microgaps and preventing bacterial microleakage [65].

Adhesive systems specifically designed for PEEK, such as primers containing methyl methacrylate, have demonstrated improved bond strengths to metals and ceramics [67]. However, the long-term durability of these adhesive bonds in the oral environment has not been systematically characterized. Hydrolytic degradation of the adhesive layer, temperature fluctuations, and cyclic loading could all compromise the adhesive bond over time.

Plasma treatment, sandblasting, and acid etching have been employed to modify the PEEK surface, increasing its surface energy and improving its adhesion to other materials [67]. These surface modifications can enhance both mechanical interlocking and chemical adhesion. However, the stability of the modified surface under oral conditions must be confirmed.

PEEK can be injection molded or overmolded onto a titanium core, creating a mechanically interlocked interface without the need for adhesives. This approach offers the advantage of producing a seamless interface, but it introduces challenges related to thermal stresses, dimensional tolerances, and the potential for degradation of the PEEK during the high-temperature processing.

Regardless of the joining method employed, several challenges remain. The interfaces must withstand cyclic loading without delamination, maintain a fluid-tight seal to prevent bacterial microleakage, and resist degradation in the presence of saliva and other oral fluids. Future research should systematically evaluate the long-term durability of Ti-PEEK and PEEK-Zr interfaces under simulated oral conditions, including thermocycling, immersion in artificial saliva, and cyclic loading.

#### 7.1.4. Implant–Abutment Connection and Insertion Torque Pathway

The design will employ an internal conical connection (e.g., Morse taper) combined with a hexagonal or octagonal internal indexation feature. This geometry offers several advantages: it provides excellent stability, facilitates a friction-lock seal, and ensures that the insertion torque is transmitted exclusively through the titanium core.

Critically, the surgical driver will engage the internal indexation feature machined directly into the solid Ti-6Al-4V core. The PEEK and zirconia layers are passive sleeves that are “pushed” into their final position by the axial force of the insertion, but they do not experience the rotational shear force of the 35–50 Ncm insertion torque. This design is intended to isolate the PEEK layer from the torque pathway, although this remains to be experimentally confirmed, preventing the deformation or adhesive failure at the Ti-PEEK interface during surgical placement, a concern we previously highlighted [65]. The mechanical interlocking features (e.g., micro-grooves) that stabilize the Ti-PEEK interface may, however, create localized stress peaks at this interface during assembly; this is precisely the type of interfacial behavior that must be modeled and validated in future FEA and experimental studies.

#### 7.1.5. Design Rationale

The design targets the cervical bottleneck, the exact locus where extreme functional loads, geometric shifts, and material discontinuities converge. Deploying a PEEK layer directly at this intersection offers a calculated structural mechanism to mitigate the abruptness of the metal-ceramic boundary. The Ti-PEEK-Zr model should not be interpreted as a simple association of materials, but as an assembly designed to influence stress distribution in a biomechanically critical region, with clear engineering specifications that are proposed to guide future validation studies.

### 7.2. Anticipated Biomechanical Advantages

The main anticipated biomechanical advantage of the Ti-PEEK-Zr concept consists of reducing the abrupt nature of the direct titanium–zirconia interface. Conventional systems force a direct and abrupt load transfer from ductile metal straight into rigid ceramic. This collision inherently concentrates stress within the brittle component, compromising the cervical zone where oblique loading generates substantial tensile and bending forces [23,28,29,30,31,32,47,49].

The introduction of an intermediate PEEK layer may modify this mechanical transfer. Due to its reduced stiffness, PEEK may allow controlled local deformation and may act as a compliant zone between two rigid components. In this way, the hypothesis of a more favorable stress distribution and a reduction in maximum values developed in the ceramic collar can be formulated.

Another anticipated advantage is related to the possibility of reducing the risk of crack initiation in zirconia. Owing to the inherently brittle nature of Y-TZP, its fracture behavior is heavily governed by localized tensile peaks and the presence of geometric or microstructural stress raisers [20,59,62]. Should the inserted PEEK film effectively curtail these tensile hot spots, the cervical zone of the implant would be expected to operate under a considerably more benign mechanical regime.

Beyond these local mechanical interactions, the Ti-PEEK-Zr paradigm enables a clear division of functional responsibilities among the constituent materials. The titanium core assumes the primary load-bearing duties. The zirconia collar ensures aesthetic integration and a favorable mucosal interface, and the intervening PEEK layer serves as a mechanical compliance mediator. This deliberate allocation of distinct structural roles constitutes one of the foundational justifications for pursuing multi-material implant architectures.

That said, scientific honesty obliges us to frame these projected gains exclusively as working hypotheses that await empirical scrutiny, rather than as established clinical truths. Indeed, the available literature indicates that polymeric additions can indeed reshape the mechanical landscape of implant assemblies, yet the magnitude and direction of this effect are profoundly contingent upon the specific device geometry, the intimacy of interfacial contacts, the precise material grade, the orientation of the applied masticatory vectors, and the quality of the underlying bone support [42,53,69]. Therefore, the benefit of the PEEK layer must be demonstrated through a numerical and experimental validation strategy.

### 7.3. Research Hypothesis

The central hypothesis of the Ti-PEEK-Zr concept is that the introduction of an intermediate compliant PEEK layer between the Ti-6Al-4V implant body and the Y-TZP cervical collar may modify stress distribution in the cervical region and may reduce local loads developed in the ceramic component.

This hypothesis rests on three key biomechanical pillars. Structurally, the cervical region acts as a natural stress amplifier under oblique loading [23,28,29,30,31,32,47,49]. Materially, fusing titanium directly to zirconia guarantees a destructive mechanical discontinuity [10,20,59,60,62,63]. Functionally, PEEK possesses the exact low-stiffness polymeric profile required to bridge this structural gap [33,34,36,39,42,53,67,69].

The hypothesis does not claim that PEEK would completely eliminate stresses developed at the interface or that it would guarantee prevention of zirconia fracture. More rigorously, the hypothesis states that the PEEK layer may modify the mode of load transfer and may reduce certain local stress concentrations, depending on assembly geometry, layer thickness, contact conditions, and loading regime.

This formulation is important for maintaining the scientific character of the concept. The Ti-PEEK-Zr system should be regarded as a proposed solution that requires validation through numerical and experimental methods, not as an already clinically confirmed solution. At this stage, the value of the concept lies in substantiating a coherent biomechanical hypothesis and in defining a research direction that can be objectively tested.

### 7.4. Need for Numerical Validation Through the Finite Element Method

Numerical validation through the finite element method represents an important stage for evaluating the Ti-PEEK-Zr concept. This method allows analysis of stress and deformation distribution in implant components and peri-implant bone, as well as identification of biomechanically critical areas [23,24,25].

To rigorously validate this concept, future numerical protocols must juxtapose at least two distinct configurations: a titanium–zirconia system without an intermediate layer and a titanium-PEEK-zirconia system with a compliant layer. This comparison would allow evaluation of the influence of PEEK on the stress field developed in the ceramic collar and in the implant–collar transition area.

The main parameters to be analyzed include von Mises equivalent stresses in metallic components, principal stresses in zirconia and PEEK, total deformations of the assembly, and stress distribution at the interfaces. In the case of zirconia, principal stresses are particularly relevant, because brittle ceramic materials are sensitive to tensile loading and crack initiation [20,59,62].

Any credible numerical assessment must incorporate off-axis loading configurations, as these produce bending moments that far more faithfully reproduce clinical failure patterns and highlight cervical zones prone to excessive stress, compared with purely vertical test conditions [43,47,49]. A growing body of recent FEA-based investigations, spanning both quasi-static and time-varying regimes, consistently advocates for a comprehensive output strategy: concurrent reporting of von Mises equivalents, principal tensile/compressive vectors, deformation fields, and projected fatigue endurance has become critical for a holistic understanding of implant-prosthetic mechanics [70].

Beyond load selection, the fidelity of any numerical model hinges on accurate assignment of material constants, faithful representation of interfacial contact behavior, the specified thickness of the polymeric buffer, and any deliberate geometric idealizations adopted to render the problem tractable. In multi-material constructs, the way in which contact between adjacent bodies is mathematically defined is paramount. The resulting predictions can shift markedly depending on whether the interfaces are assumed to be perfectly bonded, frictional, or capable of partial separation.

A particularly active frontier in contemporary computational implantology involves the cross-correlation of FEA outputs with sophisticated data-driven surrogates. The strategic coupling of finite-element predictions with artificial neural network architectures now drives the forecasting of von Mises fields, deformation metrics, and fatigue safety margins [71]. This algorithmic synergy does not merely supplement conventional mechanical analysis. It forces a definitive shift toward accelerated, predictive design optimization. Beyond forecasting, numerical simulation operates as the absolute gatekeeper in early device development. Rigorously manipulating geometric variables, specifically the PEEK interlayer thickness, the ceramic ferrule contour, and the material junction topology, mathematically isolates the structural configurations that command a demonstrably superior stress profile. However, FEA is not employed here to provide definitive clinical guarantees. Rather, it functions as the essential theoretical proving ground, furnishing the quantitative benchmarks that will subsequently govern the design of physical validation experiments.

Beyond conventional FEA, future numerical simulations should incorporate the effects of environmental factors, including temperature-dependent material properties and the influence of moisture on the mechanical behavior of PEEK. While these factors are challenging to model, they represent important variables that could significantly affect the long-term performance of the implant system.

### 7.5. Need for Experimental Validation

Experimental corroboration remains an indispensable corollary to computational prediction, serving to verify the findings derived from numerical models and to assess the actual mechanical performance of the Ti-PEEK-Zr system under stringently controlled laboratory conditions. While FEA affords invaluable insight into stress distributions, its outputs are contingent upon the modeling simplifications adopted, the property values assigned to each material, and the chosen representation of contact kinematics.

The experimental campaign should proceed in a staged fashion, beginning with quasi-static load-to-failure tests to establish the baseline strength of the assembly and to characterize the predominant failure mode, followed by cyclic fatigue protocols. The latter are essential: the oral environment continuously subjects implants to repetitive loading events, and long-term functional survival is overwhelmingly dictated by the progressive accumulation of subcritical microdamage [49,64].

Several recent investigations that have directly correlated FEA predictions with fatigue bench testing of dental implants underscore the merits of a dual-pronged strategy, using numerical modeling to pre-identify critical hotspots and then validating the system’s actual response through standardized mechanical interrogation [72]. This combined approach is particularly germane to the Ti-PEEK-Zr proposition, as it offers a direct means of determining whether the anticipated stress-attenuating effect of the PEEK interlayer is indeed manifested in the tangible behavior of the physical construct.

For the Ti-PEEK-Zr system, experimental validation must pay especially close attention to the zirconia collar and to the two critical interfaces, titanium-PEEK and PEEK-zirconia. The post-test inspection should systematically search for evidence of cracking, interfacial debonding, localized plastic yielding, loss of contact integrity, or any positional shifts in the components following mechanical challenge. These phenomena can be effectively interrogated through a suite of complementary characterization modalities, including optical microscopy, scanning electron microscopy, energy-dispersive X-ray spectroscopy, surface profilometry, and other advanced structural or morphological assessment techniques.

The assembly protocol itself warrants careful consideration. Should the PEEK layer fail to establish stable, intimate apposition, or should the interfaces exhibit adaptation imperfections, the anticipated biomechanical benefits could be substantially diminished, or even nullified. Experimental validation must unequivocally transcend the superficial extraction of global strength indices. It demands an uncompromising interrogation of interfacial integrity and structural cohesion.

Only through the systematic, iterative cross-validation of predictive FEA models, destructive mechanical testing, and high-resolution interface characterization can the protective efficacy of the PEEK layer be established. This uncompromising, staged methodology provides evidence for the only objective pathway to validate and optimize the next generation of multi-material implant architecture.

In addition to mechanical testing, a comprehensive validation framework must include biological and environmental assessments. Thermocycling and pH cycling should be employed to simulate the thermal and chemical challenges of the oral environment. Artificial saliva immersion should be used to evaluate the degradation of the PEEK layer and the stability of the interfaces under simulated oral conditions. Biofilm challenge assays, using multi-species bacterial models, should be conducted to assess the susceptibility of the materials to bacterial colonization and the risk of peri-implantitis. Microleakage assessments should be performed to confirm the integrity of the Ti-PEEK and PEEK-Zr interfaces. Finally, biological evaluation of bone and soft tissue responses should be conducted using appropriate in vitro and in vivo models to confirm the biocompatibility of the proposed implant system. Only through the integration of mechanical, biological, chemical, and microbiological assessments can the clinical feasibility of the Ti-PEEK-Zr concept be rigorously established.

## 8. Future Research Directions

### 8.1. Numerical Validation of the Ti-PEEK-Zr Concept

The immediate priority for future research is the rigorous numerical validation of the Ti-PEEK-Zr architecture via finite element analysis. This stage is necessary to evaluate how the introduction of an intermediate PEEK layer influences stress distribution in the cervical region of the implant assembly and, in particular, in the zirconia ceramic component.

Numerical analysis should begin by comparing distinct but dimensionally equivalent geometric models. A first model should represent a titanium–zirconia configuration without an intermediate layer, while a second model should include the PEEK layer positioned between the Ti-6Al-4V implant body and the Y-TZP cervical collar. Comparing these models may allow identification of the changes produced by the compliant layer on the stress and deformation fields.

The main parameters monitored should include von Mises equivalent stresses in metallic components, maximum and minimum principal stresses in zirconia, total deformations of the assembly, and stress distribution at the titanium–PEEK and PEEK–zirconia interfaces. In the case of zirconia, analysis of principal stresses is particularly important because this material has predominantly brittle behavior, and local tensile loads may promote crack initiation [20,59,62].

To ensure clinical relevance, future loading conditions must strictly adhere to the parameters outlined in the ISO 14801 standard for dynamic fatigue testing of endosseous dental implants [49]. Numerical models should implement a 30-degree load inclination off the implant axis and incorporate a 3mm nominal bone clearance to accurately replicate worst-case scenarios involving crestal bone resorption.

Several recently published computational reports have reaffirmed the discriminatory power of finite element modeling when applied to biomaterials of contrasting rigidity, notably PEEK, carbon-fiber-reinforced PEEK, titanium, and zirconia, tested under both axial and tilted occlusal forces [73,74,75]. These findings indicate that a numerical validation of the Ti-PEEK-Zr design cannot rely on a single scalar output. Instead, it demands a panoramic mechanical characterization: von Mises criteria for the metallic core, principal stress trajectories for the ceramic phase, cumulative deformation profiles, and a detailed mapping of load-transfer evolution toward the supporting bone bed must all be captured simultaneously.

Another pivotal variable meriting systematic exploration is the dimensional thickness of the polymeric buffer. An excessively thin interlayer may exert only marginal influence on force redistribution, while an overly thick one risks amplifying local deformability or compromising the overall stability of the device. The numerical campaign should encompass multiple geometric permutations, with the explicit objective of pinpointing an optimal compromise between stiffness preservation, controlled compliance, and effective shielding of the brittle ceramic member.

The mathematical representation of interfacial contact likewise warrants careful scrutiny. Numerical simulations must explicitly enforce specific boundary conditions, spanning from absolute interfacial bonding to frictional micro-separation, because these contact mechanics fundamentally dictate the resultant stress fields. Within the Ti-PEEK-Zr architecture, the strict physical cohesion among these distinct layers absolutely governs load transfer and long-term survivability.

This computational tier does not attempt to simulate clinical evidence. It operates as an aggressive theoretical sieve. It mathematically isolates structurally viable architectures long before committing resources to physical fabrication. Consequently, FEA dictates the experimental trajectory, forcing physical testing exclusively toward mechanically coherent configurations and locking down the critical parameters that control multi-material implant performance [23,24,25,70,71,72,73,74,75].

### 8.2. Experimental Validation of the Ti-PEEK-Zr Concept

Physical validation is necessary. It must follow in silico optimization without exception. Numerical models, irrespective of their mathematical refinement, remain intrinsically constrained by the assigned boundary conditions, the chosen constitutive material laws, and the necessary geometric idealizations. Physical testing is the mechanism by which these theoretical constructs are forced to confront actual mechanical behavior.

The experimental protocol must therefore operate on two distinct levels. One concerns global structural survival; the other, local interfacial cohesion. An initial quasi-static load-to-failure sequence reveals the baseline strength of the system. Equally critical, it demarcates the primary failure vector—that is, it isolates definitively whether collapse originates in the zirconia collar, the PEEK buffer, the mechanical junctions, or the titanium core.

**Figure 4:**
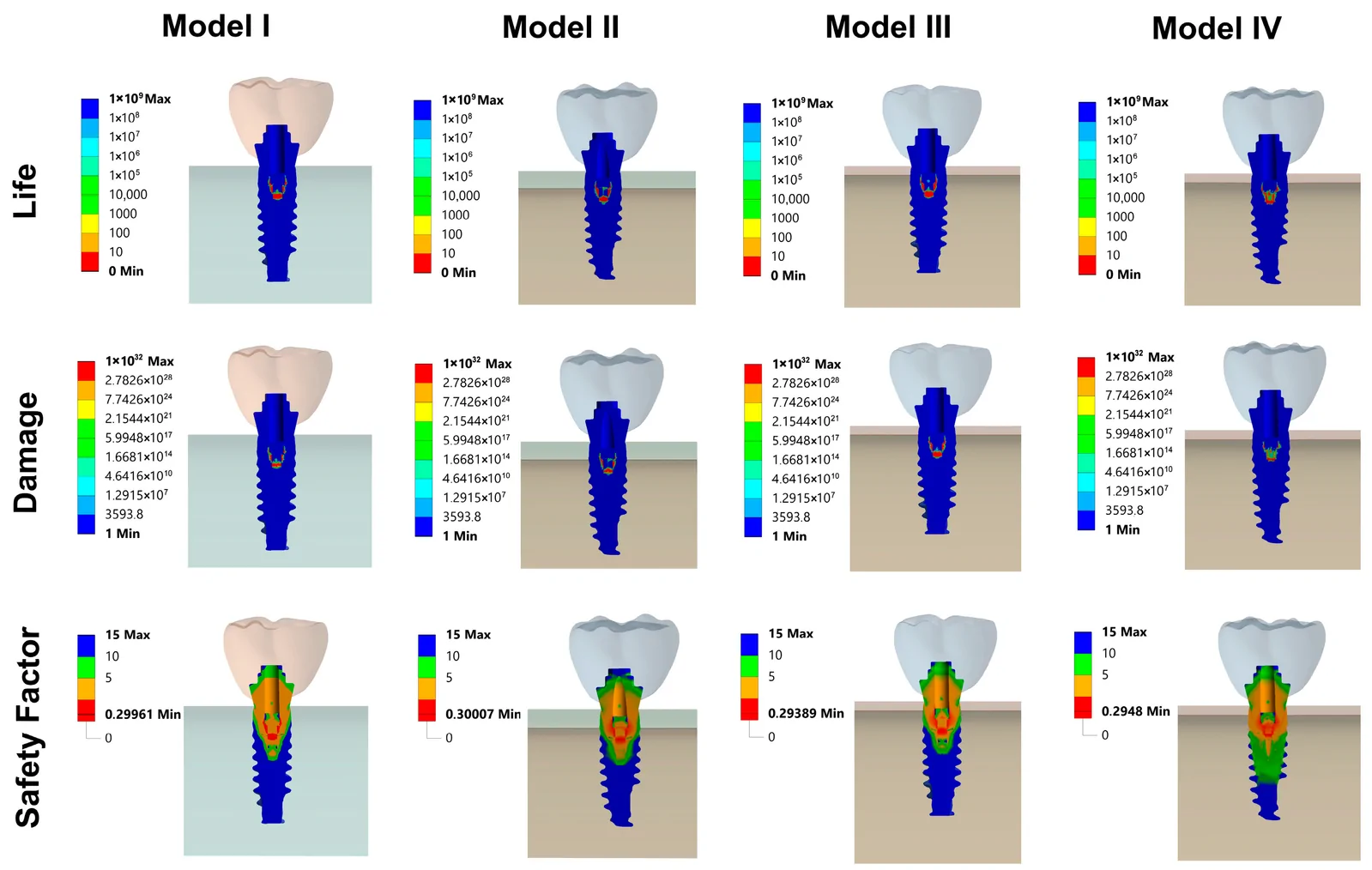
Fatigue behavior analysis projecting structural life, damage accumulation, and safety factors under cyclic functional loading (adapted with permission from [53]).

Yet, static failure mapping is fundamentally incomplete. Dynamic cyclic fatigue testing must subsequently take absolute precedence. The oral cavity imposes relentless cyclic loading; monotonic data, in this context, is critically insufficient for any meaningful long-term prognosis. Only dynamic interrogation systematically uncovers the progressive deterioration mechanisms, crack nucleation within the ceramic ferrule, viscoelastic creep affecting the PEEK stratum, and the gradual degradation of multi-material interfaces [49,64,72]. These theoretical fatigue limits are not abstract projections. Figure 4 [53] translates them directly into concrete damage accumulation forecasts. The conclusion remains unequivocal: exclusive reliance on static strength metrics fundamentally underestimates the long-term structural decay of the system.

Component examination following mechanical tests must be a necessary procedural step. Several analytical techniques are available for this purpose: optical microscopy, scanning electron microscopy (SEM), energy-dispersive X-ray spectroscopy (EDS), and surface profilometry. Together, they yield substantial information on contact surface condition, the presence of wear scars, incipient microcracks, localized debonding, plastic deformation, and other morphological alterations induced by the applied mechanical challenge. This stage carries particular weight. Multi-material constructs frequently exhibit failure modes that elude detection through global strength metrics alone.

The combined use of mechanical testing and numerical simulation is becoming more widely adopted in implant evaluation. Recent investigations into additively manufactured zirconia components have demonstrated the value of pairing mechanical assays with finite element analysis. This combination has proven effective in locating stress concentration hotspots and identifying potential fracture origins [76]. That same synergistic rationale can be extended to the Ti-PEEK-Zr configuration, with particular relevance for detailed inspection of the ceramic collar and the adjacent material junctions.

Manufacturing and assembly protocols carry equal significance. Reproducible contact conditions demand that the titanium body, the PEEK interlayer, and the zirconia collar each be produced within strictly defined dimensional tolerances. If interfacial mismatch occurs, regardless of its magnitude, it has the potential to alter the intended stress field and, in doing so, compromise the interpretation of results obtained from mechanical testing. Experimental validation, therefore, must be closely coordinated with thorough geometric and morphological characterization of test specimens, performed both before and after loading.

To close the validation loop, a systematic correlation must be established between the fracture patterns observed in physical specimens and the predictions generated by FEA. Only through this iterative cross-validation between computational models and experimental data can the Ti-PEEK-Zr system move beyond theoretical conjecture and achieve the status of a validated structural solution.

### 8.3. Perspectives for Optimizing Multi-Material Implant Design

Optimization of the Ti-PEEK-Zr architecture demands an incremental, feedback-driven approach. It requires the strict integration of three complementary data sources: numerical simulation, mechanical testing, and interfacial characterization. The optimization agenda is twofold. Achieving global strength is necessary, yet insufficient on its own. Localized stresses within the ceramic must be actively curtailed, interface stability preserved, and excessive deformation of the compliant buffer rigorously constrained.

Geometric refinement must initially target the PEEK stratum. Its thickness, contour, and spatial continuity directly dictate the biomechanical response of the entire assembly. A layer that proves too thin exerts negligible influence on force transmission. At the opposite extreme, excessive bulk amplifies local deformability and directly jeopardizes the ceramic collar’s stability. The engineering objective is a configuration that facilitates stress redistribution without compromising the functional rigidity of the overall construct.

Subsequent adjustments must address the zirconia collar. Given the elastic-brittle nature of Y-TZP, the design must scrupulously avoid geometric stress raisers. Sharp corners are strictly inadmissible. Furthermore, abrupt sectional transitions and excessively slender, heavily loaded zones must be completely eliminated. The collar geometry requires rational coordination with the PEEK buffer’s position and the anticipated trajectory of occlusal forces.

Stabilizing the titanium-PEEK and PEEK-zirconia interfaces is equally critical. In multi-material systems, overarching performance hinges not merely on bulk constituent properties, but decisively on the quality of physical contact across each junction. Interface optimization encompasses surface roughening, mechanical interlocking matrices, chemical functionalization, or micro-retentive architectures. Recent inquiries into PEEK surface modification confirm that augmenting interfacial behavior constitutes a primary trajectory for biomedical applications [67,68].

Emerging evidence regarding PEEK further highlights the pivotal role of processing parameters, additive manufacturing fidelity, and porosity management [68]. These variables are highly consequential for the Ti-PEEK-Zr system. The functional viability of the interlayer depends heavily on manufacturing precision and interfacial adaptation, not just its baseline elastic modulus.

Contemporary implant design must pivot toward integrated predictive frameworks. Finite element analysis serves as more than a validation tool for a final configuration; it is an essential screening mechanism for evaluating geometric variants in silico prior to physical fabrication. Fusing FEA with advanced predictive algorithms accelerates this optimization cycle, rapidly isolating configurations with diminished mechanical risk profiles [72].

Ultimately, the Ti-PEEK-Zr paradigm establishes a versatile research platform for multi-material dental implants, wherein each constituent fulfills a demarcated functional niche. Titanium delivers primary load-bearing capacity; zirconia ensures aesthetic harmony and mucosal integration; PEEK functions as the biomechanical compliance mediator. This philosophy shifts the developmental focus from mere biocompatibility toward the active structural management of stress distributions.

Advancing this paradigm demands a relentless, sequential methodology: theoretical substantiation, destructive physical testing, and continuous interfacial optimization. Only through the systematic correlation of these three evidentiary tiers can we determine the biomechanical advantage of the PEEK layer within titanium–zirconia implant systems.

## 9. Limitations of the Present Review

This is a narrative review, not a formal systematic meta-analysis. The selection of sources was deliberately oriented toward studies offering mechanistic support for the Ti-PEEK-Zr rationale. Strict inclusion criteria were avoided precisely to preserve the theoretical breadth required for this interdisciplinary synthesis.

The current published record contains no dedicated experimental or clinical investigations of this specific Ti-PEEK-Zr tri-layered construct. What we advance here is an argument built upon the integration of disparate data, titanium, zirconia, PEEK, multi-material implantology, finite-element modeling, and dental implant mechanical testing. Our conclusions are best understood as well-substantiated biomechanical hypotheses. They are not, and should not be misconstrued as, direct evidence of clinical efficacy.

A critical limitation of the current theoretical framework is that findings from studies involving PEEK abutments, CFR-PEEK implants, polymeric attachments, or alternative implant geometries should not be directly extrapolated to the behavior of the proposed tri-layer implant. We acknowledge this constraint explicitly: these data were used solely to illustrate generic buffering potential and to define the critical parameters for future validation studies, not to provide evidence of efficacy for our specific configuration.

Translating this theoretical model into tangible clinical application introduces additional constraints. Numerous physical variables must be controlled, yet literature synthesis alone cannot adequately capture them. Device geometry, PEEK layer thickness, surface roughness and treatment protocols, interfacial contact quality, assembly methodology, manufacturing tolerances, and the applied loading regime, all of these factors can profoundly influence stress distribution, interface stability, and fatigue performance. Each, in isolation or combination, has the capacity to alter the mechanical response of the system.

The conceptual character of this review is the very source of its defining boundary. The contribution does not reside in demonstrating the effectiveness of a pre-validated system. It lies instead in articulating a reasoned investigative trajectory. That trajectory should now be pursued through numerical modeling, static and cyclic mechanical testing, and comprehensive morphological characterization of material interfaces, a streamlined, cost-efficient experimental sequence capable of revealing critical biomechanical insights.

Furthermore, the present review is predominantly focused on the mechanical aspects of the Ti-PEEK-Zr concept. The biological, chemical, and microbiological requirements for a successful transmucosal dental implant have not been exhaustively addressed. Surface chemistry, wettability, protein adsorption, soft tissue sealing, bacterial colonization, corrosion resistance, and cleanability are equally important for long-term implant performance. While we have highlighted these considerations as areas requiring future investigation, the current review does not provide direct experimental evidence on these topics. This limitation underscores the need for a comprehensive, multi-disciplinary approach to validating the proposed concept.

## 10. Conclusions

Designing multi-material implant systems has transitioned from an optional refinement to an important structural consideration. The strategic integration of titanium and zirconia exploits a deliberate material synergy. Titanium provides mechanical robustness, fracture toughness, and a proven load-bearing core, while Y-TZP zirconia dictates aesthetic harmony, chemical inertness, and mucosal integration.

This binary architecture, however, harbors a fundamental biomechanical vulnerability. It forces a direct structural conflict, rooted in the stark incongruity between their inherent stiffness profiles and failure modalities. This disparity assumes critical significance within the implant’s cervical region, where oblique loading, geometric discontinuities, and structural transitions converge to promote stress concentration.

To address this inherent limitation, the Ti-PEEK-Zr concept mandates the introduction of an intermediate PEEK layer between the Ti-6Al-4V core and the Y-TZP cervical collar. The role of PEEK should not be interpreted as a simple intermediate stiffness step between titanium and zirconia, because its elastic modulus is lower than that of both materials. More rigorously, PEEK may be considered a compliant layer or a functional transition element capable of locally modifying load transfer at the metal–ceramic interface.

The main conceptual contribution of the Ti-PEEK-Zr system consists of the functional separation of material roles within the same implant assembly. Titanium provides the main load-bearing support, zirconia contributes to the aesthetic and biological integration of the cervical region, and PEEK may influence the mechanical behavior of the transition zone. Introducing a PEEK interlayer may potentially disrupt this structural conflict. Acting as an elastic buffer, the polymer dampens load transfer, deliberately shielding the vulnerable ceramic collar from peak stress concentrations.

The mechanical superiority of this tri-layered architecture remains a theoretically grounded hypothesis that now demands rigorous empirical execution. The purpose of this synthesis is not to bypass physical testing, but to dictate its precise trajectory. Translating this concept into a functional clinical device hinges entirely on a structured, biphasic validation loop.

Execution begins in silico. High-resolution finite element analysis (FEA) is imperative to map von Mises stresses, principal stress distributions, and interfacial strain across the Ti-PEEK-Zr assembly. Yet computational modeling cannot stand alone. It must be immediately subjected to physical validation through static over-load and dynamic cyclic fatigue testing. Only this direct experimental sequence can definitively reveal the structural integrity, long-term survivability, and ultimate failure modes of the system. Complementarily, morphological and structural analysis using SEM, EDS, and profilometry may contribute to interface characterization and to the identification of possible local degradation or debonding mechanisms.

It is important to emphasize that the Ti-PEEK-Zr concept presented here is a biomechanical hypothesis that requires rigorous empirical validation. The mechanical superiority of the tri-layered architecture remains to be demonstrated through FEA and experimental testing. Furthermore, the biological feasibility of the concept must be confirmed through comprehensive in vitro and in vivo studies that address osseointegration, soft tissue sealing, bacterial colonization, and long-term stability in the oral environment. The authors explicitly acknowledge that the current work does not provide clinical evidence and that the concept should be interpreted as a research direction rather than a validated clinical solution.

The Ti-PEEK-Zr concept establishes a proposed structural framework designed to mitigate cervical stress concentration. Its scientific value lies in engineering a rational, testable solution to a critical biomechanical vulnerability in modern titanium–zirconia assemblies. Forthcoming numerical and physical trials will quantify the capacity of the PEEK layer to limit structural degradation and optimize multi-material implant design.

## Figures and Tables

**Table 1:** Key factors amplifying cervical stress in hybrid dental implants and their structural implications.

Biomechanical Factor	Mechanism of Stress Amplification	Primary Structural Consequence	Key References
Oblique/Non-Axial Loading	Generates bending moments and asymmetric force distribution across the implant-bone interface.	High tensile stress accumulation at the cervical boundary.	[23,25,31,32,46,49]
Local Geometric Variations	Sudden cross-sectional changes and specific abutment connections act as force bottlenecks.	Localized stress overloads independent of material properties.	[28,29,30,31,32,44]
High Stiffness Gradient (Ti vs. Zr)	Direct contact between elastic–plastic (Ti) and elastic brittle (Zr) materials forces discontinuous load transfer.	Elevated risk of crack initiation and propagation within the ceramic collar.	[10,20,47]

**Table 2:** Comparative mechanical properties of the analyzed materials.

Property	Ti-6Al-4V	Y-TZP Zirconia	PEEK
Material type	Metallic alloy	Oxide ceramic	Thermoplastic polymer
Elastic modulus	~105–115 GPa	~190–210 GPa	~3–4 GPa
Mechanical strength	High	High, but brittle	Moderate
Mechanical behavior	Elastic–plastic	Elastic-brittle	Elastic–plastic/viscoelastic
Fracture toughness	High	Low–moderate	Variable
Sensitivity to cracks	Low	High	Moderate
Role in the system	Load-bearing implant body	Cervical collar	Intermediate compliant layer
Biomechanical relevance	Takes up the main loads	May concentrate stresses	May redistribute stresses
References	[4,5,58]	[10,20,59,60,62,63]	[33,34,36,39,42]

Note: The values presented are indicative and may vary depending on composition, processing, heat treatments, degree of sintering, reinforcement, manufacturer, and testing methodology.

**Table 3:** Comparative peak von Mises stress values (MPa) between monolithic titanium and PEEK-integrated models under oblique loading (adapted from [42], open access under CC BY 4.0 license).

Component	Titanium Model (MPa)	PEEK Model (MPa)	Stress Variation (%)
Abutment	390.4	339.2	−13.1%
Fixation Screw	305.6	470.6	+53.9%
Implant Fixture	335.6	868.1	+158.6%

## Data Availability

No new data were created or analyzed in this study. Data sharing is not applicable to this article.
